# CT Atlas of the Coelomic Cavity in the Yellow-Legged Gull (*Larus michahellis*)

**DOI:** 10.3390/ani16152438

**Published:** 2026-08-06

**Authors:** Jose Raduan Jaber, Alvaro Ros, Yareli Rodriguez, Pablo Paz-Oliva, Magnolia Conde-Felipe, Conrado Carrascosa, Alejandro Morales-Espino

**Affiliations:** 1 Departamento de Morfología, Facultad de Veterinaria, Universidad de Las Palmas de Gran Canaria, Trasmontaña, Arucas, 35413 Las Palmas de Gran Canaria, Spain; alvaro.ros101@alu.ulpgc.es (A.R.); yareli.rodriguez102@alu.ulpgc.es (Y.R.); pablo.paz101@alu.ulpgc.es (P.P.-O.); 2 Grupo de Innovación Educativa Aplicada a la Docencia Veterinaria (VETFUN), Universidad de Las Palmas de Gran Canaria, 35001 Las Palmas de Gran Canaria, Spain; mmconde@uco.es (M.C.-F.); conrado.carrascosa@ulpgc.es (C.C.); 3 Departamento de Sanidad Animal, Universidad de Córdoba, 14071 Córdoba, Spain; 4 Departamento de Patología y Tecnología de los Alimentos, Facultad de Veterinaria, Universidad de Las Palmas de Gran Canaria, 35413 Las Palmas de Gran Canaria, Spain; 5 IVC Evidensia Los Tarahales, 35013 Las Palmas de Gran Canaria, Spain; alejandro.morales108@alu.ulpgc.es

**Keywords:** anatomical sections, coelomic cavity, computed tomography, seabirds, yellow-legged gull

## Abstract

The Yellow-legged Gull (*Larus michahellis*) is one of the most frequently admitted seabirds to wildlife rehabilitation centers in the Mediterranean and Atlantic regions. Despite its clinical relevance, detailed computed tomographic references of the coelomic cavity are lacking. The aim of this study was to describe the normal cross-sectional anatomy and computed tomographic appearance of the coelomic cavity in this species and to develop an anatomical atlas to facilitate diagnostic image interpretation. Eight young adult female Yellow-legged Gull were examined post-mortem. Six specimens underwent computed tomography using a 16-slice helical scanner, followed by frozen transverse sectioning, while two specimens were dissected to provide detailed anatomical correlation. CT datasets were evaluated using bone, soft tissue, and pulmonary window settings, and three-dimensional volume-rendered reconstructions were generated. Anatomical dissections, cross-sectional slices, and CT images enabled identification and characterization of the principal structures of the respiratory, cardiovascular, digestive, urinary, and reproductive systems, as well as their spatial relationships within the coelomic cavity. The combined evaluation of gross anatomy and CT imaging allowed accurate recognition of major organs, air sacs, large vessels, and skeletal landmarks throughout the examined sections. The atlas provides a comprehensive reference for normal coelomic anatomy in *Larus michahellis* and establishes a baseline for the interpretation of CT examinations in clinical, rehabilitation, and research settings. These findings may improve diagnostic accuracy and support future investigations of coelomic disorders in this species.

## Simple Summary

The Yellow-legged Gull (*Larus michahellis*) is a common seabird frequently treated in wildlife rehabilitation centers, where injuries and diseases affecting the coelomic cavity are regularly encountered. Computed tomography (CT) is increasingly used in avian medicine because it provides detailed images of internal structures without the superimposition commonly seen in conventional radiography. However, interpretation of CT studies requires accurate knowledge of normal anatomy, and no CT atlas is currently available for this species. In this study, CT examinations, anatomical dissections, and cross-sectional sections were combined to describe the normal anatomy of the coelomic cavity in adult Yellow-legged Gulls. Major organs, air sacs, blood vessels, and skeletal structures were identified and correlated with their CT appearance. The resulting atlas provides a reference for veterinarians, wildlife clinicians, and researchers, facilitating the interpretation of CT images and improving the diagnosis of coelomic diseases in this species.

## 1. Introduction

Yellow-legged gulls (*Larus michahellis*) are among the most frequently encountered free-ranging seabirds in Mediterranean–Atlantic veterinary practice and are common patients in wildlife rehabilitation centers. In a long-term Gran Canaria series, Yellow-legged Gulls represented 46.52% of admitted seabirds, with light pollution, intoxication, and trauma among the leading causes of morbidity [1]. This sustained clinical exposure gives clear practical relevance to diagnostic imaging in Laridae, particularly when external examination underestimates intracoelomic disease or multisystem involvement in debilitated seabirds. Moreover, gulls occupy a synanthropic ecological niche at the interface of marine, urban, and waste-associated environments, which heightens the importance of precise diagnosis in both clinical and epidemiological terms [1,2].

In birds, the thoracic and abdominal viscera are not completely separated as in mammals; instead, most major organs are contained within a single coelomic cavity, whose spatial organization is closely linked to fixed lungs, extensive air sacs, and highly compact visceral packing [3]. This anatomical arrangement means that even relatively subtle abnormalities, including organomegaly, displacement, effusion, granulomatous change, foreign bodies, or masses, may alter multiple organ relationships and compromise respiration, gastrointestinal transit, or renal function. Radiography remains the most accessible first-line imaging modality in avian medicine, but interpretation of the coelom is often limited by superimposition and variable serosal detail. Ultrasonography is useful for selected fluid-filled or peripherally accessible structures, yet its reach is inherently partial in birds. By contrast, computed tomography (CT) provides cross-sectional and multiplanar assessment without superimposition and, in modern avian atlases, can also provide attenuation data in Hounsfield units and morphometric reference values, thereby strengthening anatomical interpretation and lesion localization [4,5,6,7].

Despite these advances, the avian CT literature remains taxonomically uneven. Species-specific coelomic CT studies are available for pet parrots, market-age Pekin ducks, juvenile Atlantic puffins, and juvenile Cory’s shearwaters [3,4,7,8]. In contrast, CT publications in the Yellow-legged Gull appear, at present, to be restricted to a nasal cavity study with anatomical correlation and an isolated shoulder trauma case report [9,10]. Accordingly, the coelomic cavity of *Larus michahellis* remains insufficiently documented in the indexed veterinary imaging literature reviewed for this manuscript. This gap is not trivial, because extrapolation from psittacines, domestic waterfowl, or even other seabirds may be anatomically useful but not fully species-appropriate for Laridae [10,11].

Against this background, the aim of this study was to describe the normal coelomic anatomy of the Yellow-legged Gull (*Larus michahellis*) through anatomical dissections, transverse cross-sections, and computed tomography imaging, and to develop a species-specific anatomical atlas for CT interpretation. Emphasis was placed on the identification of major coelomic organs and their anatomical relationships to facilitate the interpretation of diagnostic images. In addition to filling an important gap in species-specific anatomical knowledge, this atlas may serve as a reference for clinical, anatomical, and comparative studies involving gulls and other marine avian species.

## 2. Materials and Methods

### 2.1. Animals

Eight yellow-legged gulls (*Larus michahellis*) were included in this study. The specimens had a mean body mass of 0.7451 kg ± 0.115 kg (range: 0.6139–0.9574 kg) and a mean body length of 43.75 cm, measured from the tip of the beak to the base of the tail (range: 39.5–54.5 cm). Individual morphometric data are summarized in Table 1.

Six carcasses were frozen shortly after death and stored for four days prior to computed tomography (CT) examination. Following CT acquisition, these specimens underwent necropsy. The remaining two specimens were dissected immediately post-mortem to expose the coelomic cavity and document organ topography, providing anatomical references for the identification and interpretation of the CT images. Sex was determined in all specimens by direct examination of the gonads during the post-mortem examination. All individuals were identified as females.

The birds were provided by the Consejería del Área de Medio Ambiente, Clima, Energía y Conocimiento, Cabildo de Gran Canaria (Las Palmas, Spain), after being found dead following a grounding event associated with light-pollution-induced disorientation. Ethical approval was not required because the study was conducted exclusively on carcasses, and no procedures involving live gulls were performed. The Cabildo de Gran Canaria authorized the use of the specimens for scientific and educational purposes.

### 2.2. CT Study

For CT evaluation, the specimens were thawed at room temperature for 12 h prior to scanning. No water bath or other thawing equipment was used. The specimens were simply placed in a tray and left to thaw naturally under ambient laboratory conditions until completely defrosted. Transverse sequential images were acquired using a 16-slice helical CT scanner (Astelion, Canon Medical Systems^®^, Tokyo, Japan). The birds were positioned symmetrically in dorsal recumbency on the scanning table, with a craniocaudal acquisition direction. A standardized protocol was applied with the following parameters: 120 kVp, 80 mA, a 512 × 512 acquisition matrix, a field of view of 1809 × 858, a pitch of 0.94, and a gantry rotation time of 1.5 s. Images were reconstructed with a slice thickness of 0.6 mm. To optimize the visualization of anatomical structures, different window settings were applied by adjusting window width (WW) and window level (WL), including bone (WW = 1500; WL = 300), soft tissue (WW = 248; WL = 123), and lung (WW = 1400; WL = −500) windows. No relevant differences in CT attenuation or anatomical appearance were observed within the coelomic cavity among specimens. In addition, the acquired datasets were used to generate three-dimensional volume-rendered reconstructions in standard DICOM format using OsiriX MD v.14.0 (Pixmeo SARL, Geneva, Switzerland).

### 2.3. Anatomical Sections

Following the CT examination, the carcasses were frozen at −80 °C for 72 h in an ultra-low temperature freezer (SANYO Electric Co., Ltd., Osaka, Japan) before anatomical sectioning. Subsequently, the specimens were sectioned using an electric band saw to obtain parallel slices approximately 1 cm thick. The resulting sections were gently rinsed with water to remove artifacts, such as feathers, which were carefully extracted using Adson forceps. Each section was then anatomically identified and photographed from both sides.

### 2.4. Anatomical Evaluation

For the identification and labeling of cross-sectional anatomy in correlation with the corresponding CT images, reference materials including specialized textbooks and peer-reviewed articles on avian anatomy were consulted [12,13,14,15,16,17].

In addition, anatomical specimens provided by the Department of Morphology were examined to support a detailed interpretation of coelomic structures. These materials contributed to improving the accuracy of anatomical identification and to a better understanding of the spatial organization of the coelomic cavity.

## 3. Results

**Figure 1:**
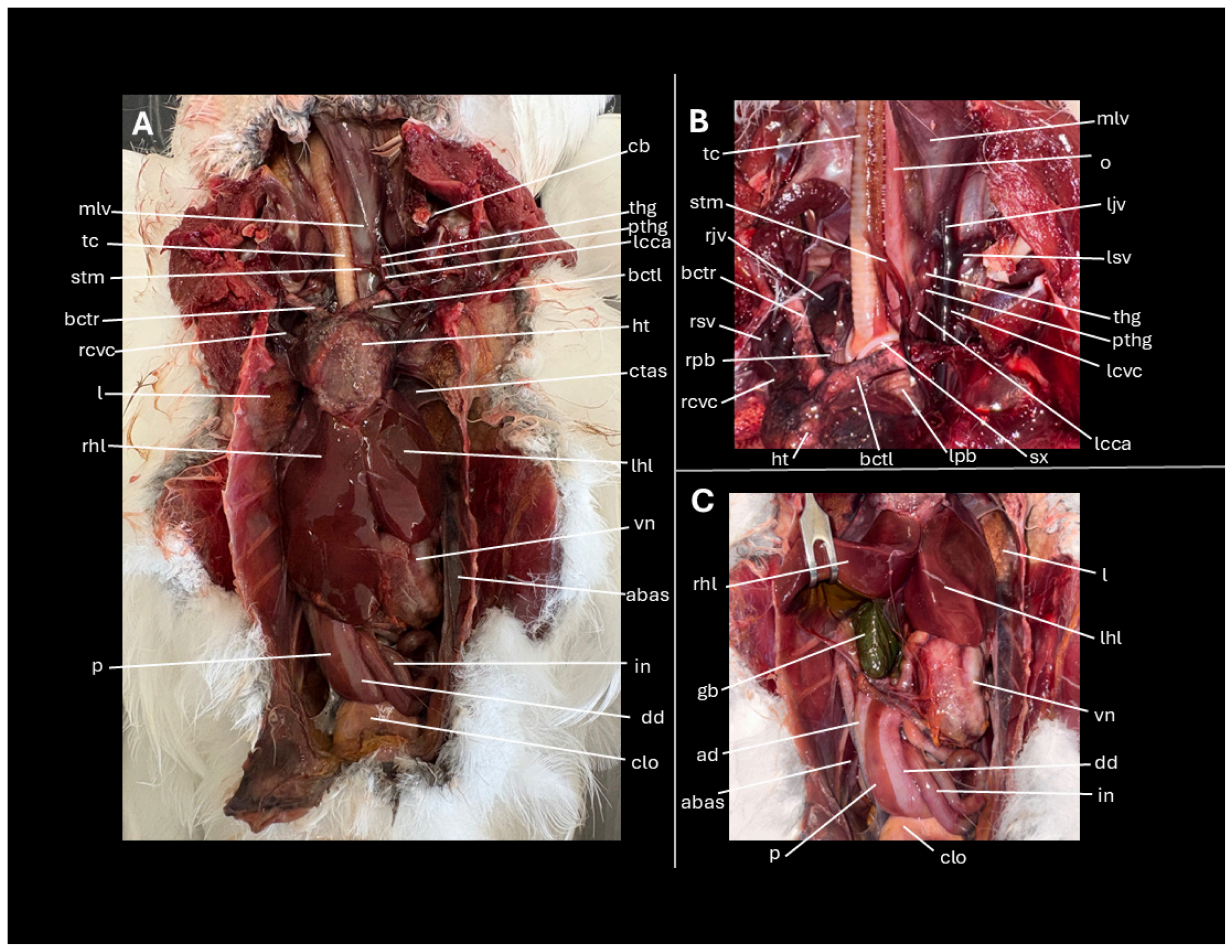
Gross dissection image of the coelomic cavity of the *Larus michahellis* (**A**), and images of the cardiovascular (**B**) and digestive (**C**) structures. abas: abdominal air sac; ad: ascending duodenum; bctl: left brachiocephalic trunk; bctr: right brachiocephalic trunk; cb: coracoid bone; clo: cloaca; ctas: cranial thoracic air sac; dd: descending duodenum; gb: gallbladder; ht: heart; in: intestine; l: lung; lcca: left common carotid artery; lcvc: left cranial vena cava; lhl: left hepatic lobe; ljv: left jugular vein; lpb: left primary bronchus; lsv: left subclavian vein; mlv: *M*. *longus colli ventralis*; o: oesophagus; p: pancreas; pthg: parathyroid gland; rcvc: right cranial vena cava; rhl: right hepatic lobe; rjv: right jugular vein; rpb: right primary bronchus; rsv: right subclavian vein; stm: sternotracheal muscle; sx: syrinx; tc: trachea; thg: thyroid gland; vn: ventriculus.

**Figure 2:**
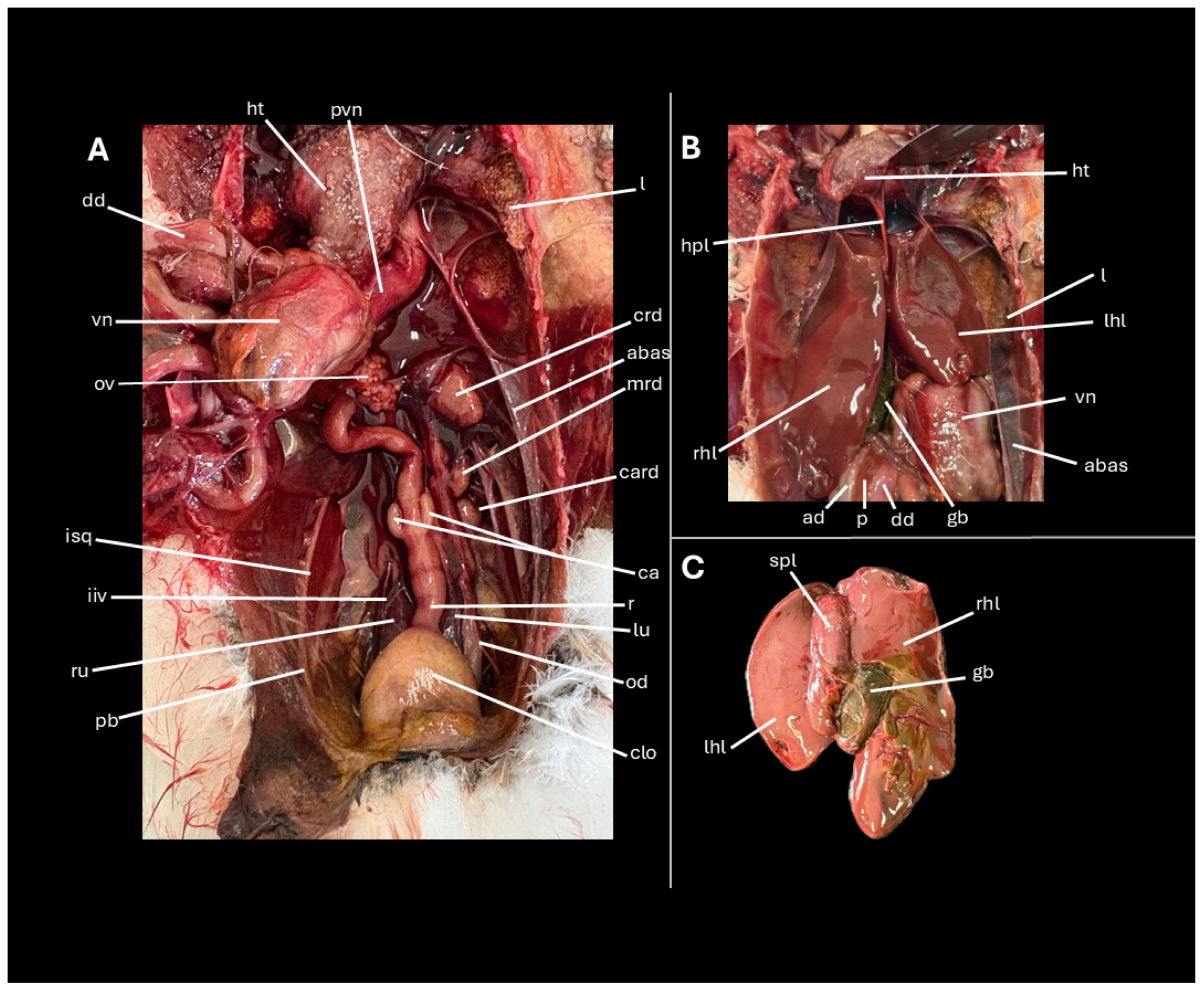
Anatomical dissection illustrates the coelomic cavity of the *Larus michahellis* after removing the liver (**A**), and magnified images showing the parietal (**B**) and visceral (**C**) surfaces of the liver. abas: abdominal air sac; ad: ascending duodenum; ca: caecums; card: caudal renal division; clo: cloaca; crd: cranial renal division; dd: descending duodenum; gb: gallbladder; hpl: hepatopericardial ligament; ht: heart; iiv: internal iliac vessels; isq: isquion; l: lung; lhl: left hepatic lobe; lu: left ureter; mrd: middle renal division; od: left oviduct; ov: left ovary; p: pancreas; pb: pubis; pvn: proventriculus; r: rectum; rhl: right hepatic lobe; ru: right ureter; spl: spleen; vn: ventriculus.

**Figure 3:**
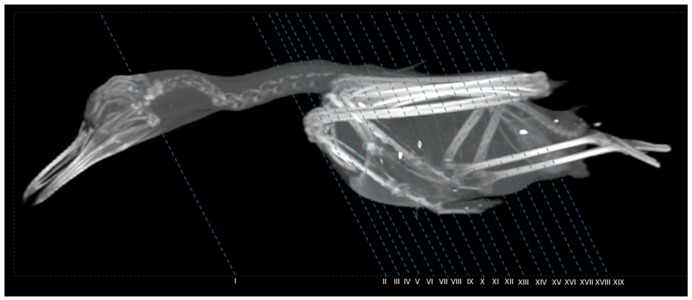
Sagital MPR volume rendering image of the body of an Yellow-legged Gull. The lines and numbers (I–XIX) represent the approximate level of the following transverse cross-sections and CT images.

**Figure 4:**
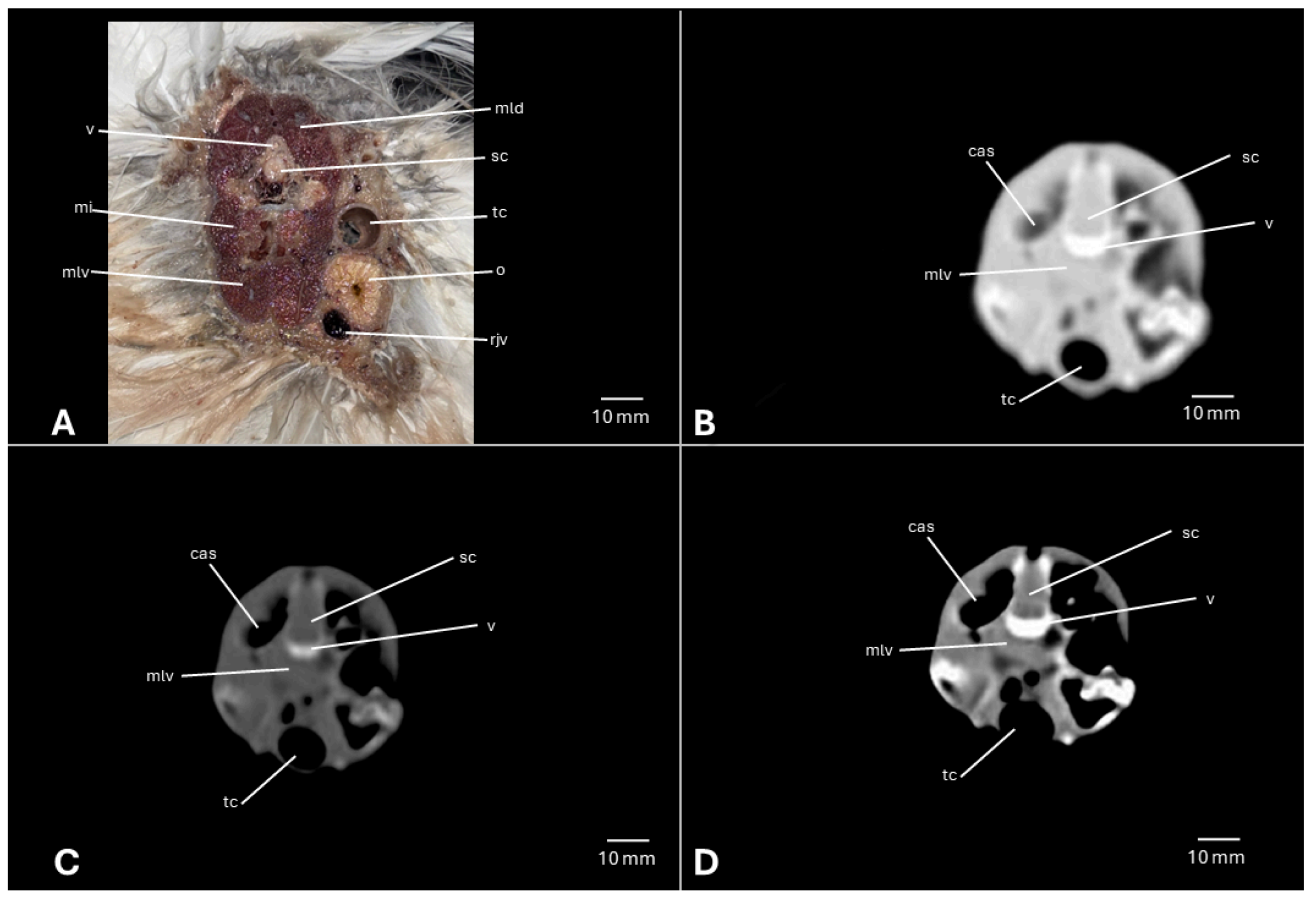
Transverse cross-sectional image (**A**), and pulmonary (**B**), bone (**C**) and soft tissue (**D**) CT images of the coelomic cavity of the *Larus michahellis* corresponding to line I in Figure 3. cas: cervical air sac; mi: *M. intertransversarii*; mld: *M. longus colli dorsalis*; mlv: *M*. *longus colli ventralis*; o: oesophagus; rjv: right jugular vein; sc: spinal cord; tc: trachea; v: vertebrae.

**Figure 5:**
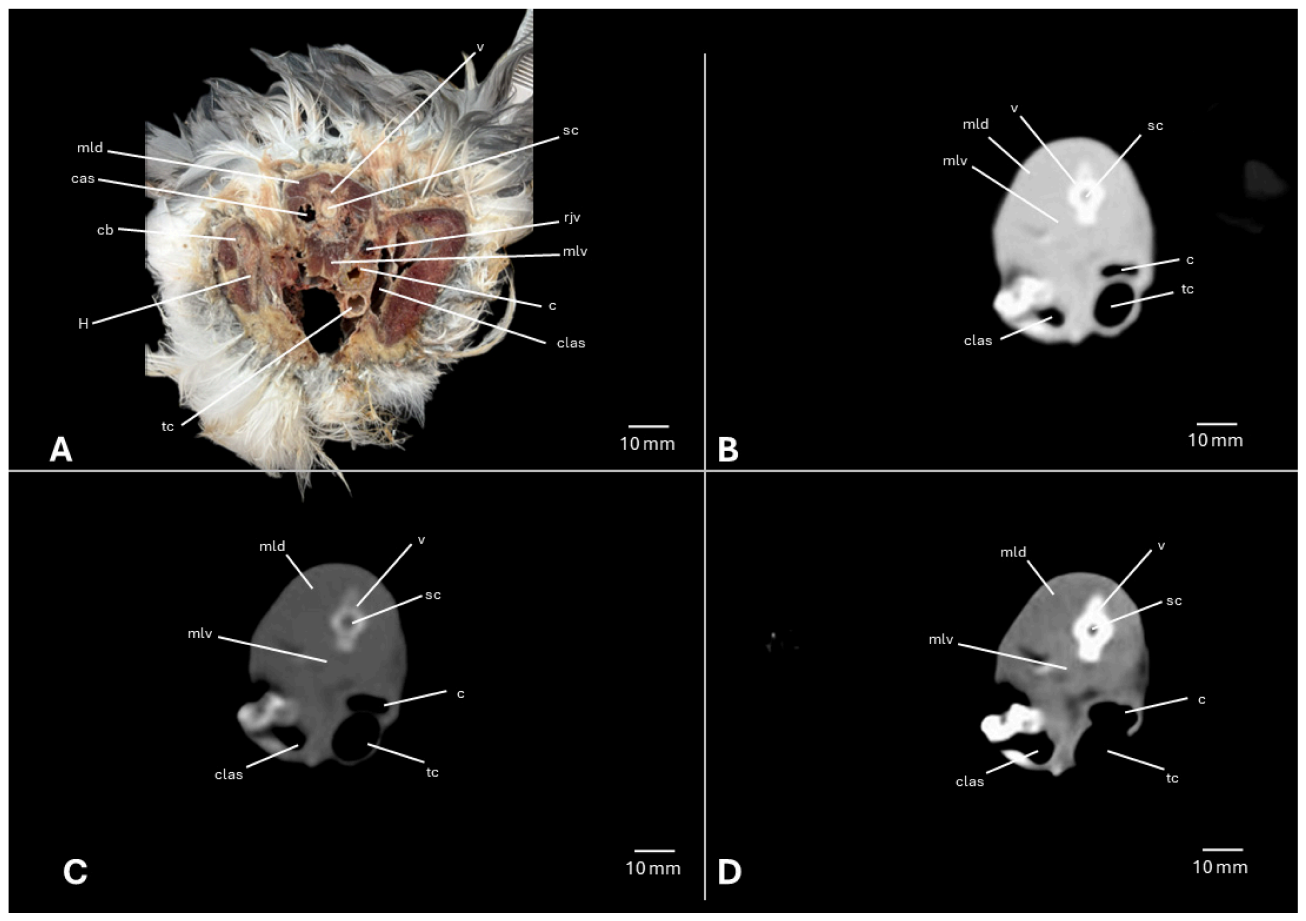
Transverse cross-sectional image (**A**), and pulmonary (**B**), bone (**C**) and soft tissue (**D**) CT images of the coelomic cavity of the *Larus michahellis* corresponding to line II in Figure 3. c: crop; cas: cervical air sac; cb: coracoid bone; clas: clavicular air sac; H: humerus; mld: *M. longus colli dorsalis;* mlv: *M*. *longus colli ventralis*; rjv: right jugular vein; sc: spinal cord; tc: trachea; v: vertebrae.

**Figure 6:**
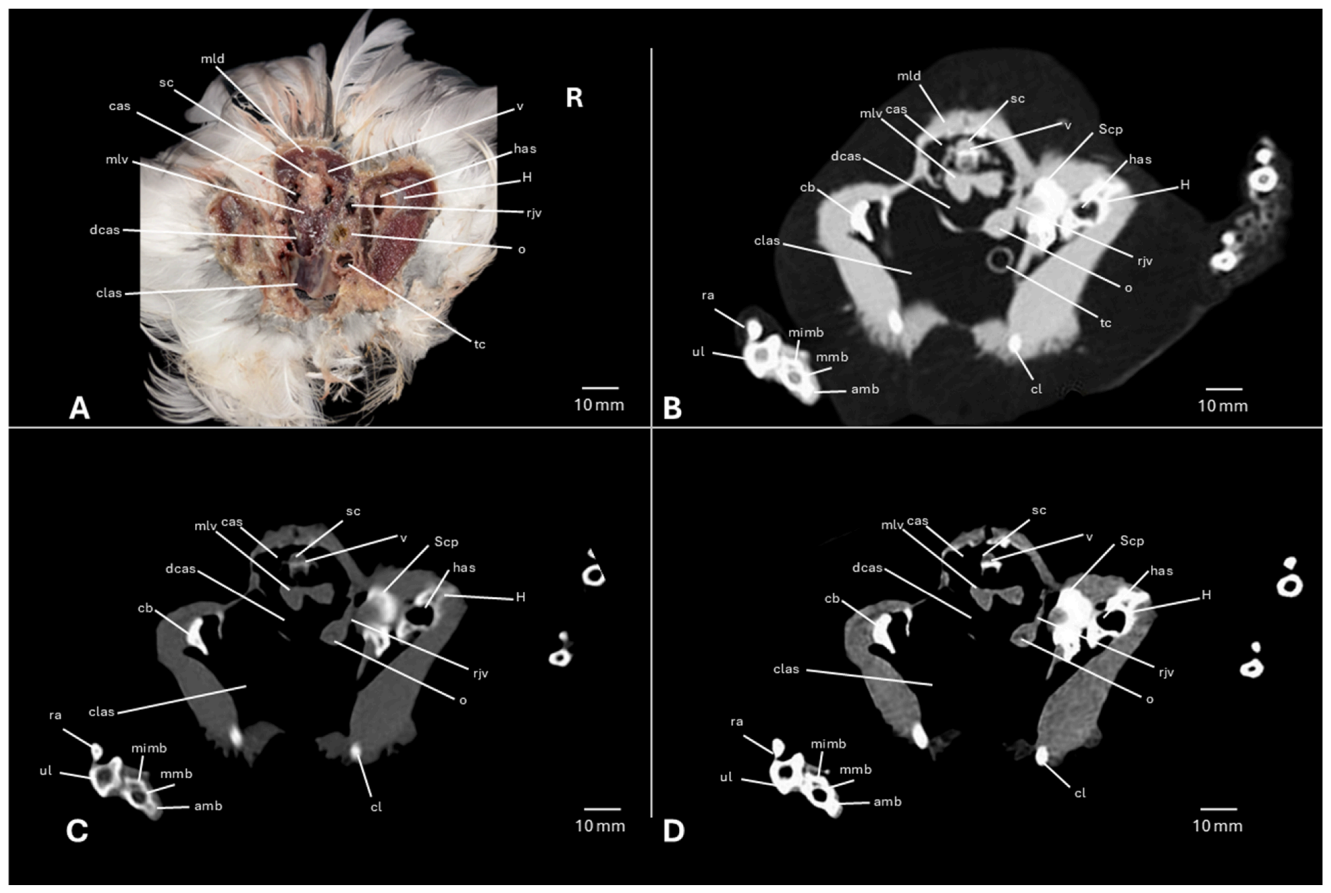
Transverse cross-sectional image (**A**), and pulmonary (**B**), bone (**C**) and soft tissue (**D**) CT images of the coelomic cavity of the *Larus michahellis* corresponding to line III in Figure 3. amb: alular metacarpal bone; cas: cervical air sac; cb: coracoid bone; cl: clavicle; clas: clavicular air sac; dcas: intrathoracic diverticula of clavicular air sac; H: humerus; has: humeral air sac; mld: M. longus colli dorsalis; mlv: M. longus colli ventralis; mmb: major metacarpal bone; mimb: minor metacarpal bone; o: oesophagus; ra: radius; rjv: right jugular vein; sc: spinal cord; Scp: scapulla; tc: trachea; ul: ulna; v: vertebrae.

**Figure 7:**
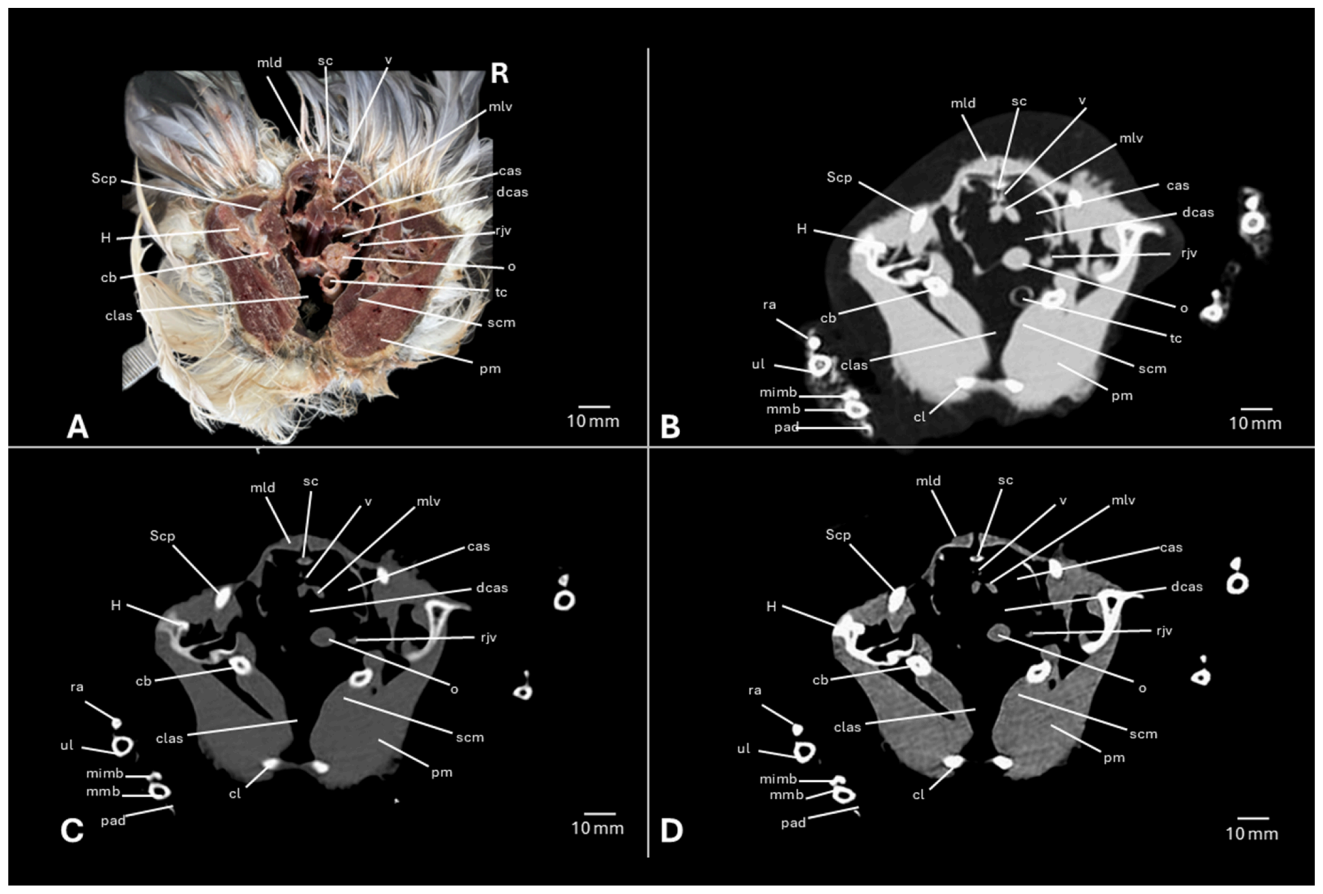
Transverse cross-sectional image (**A**), and pulmonary (**B**), bone (**C**) and soft tissue (**D**) CT images of the coelomic cavity of the *Larus michahellis* corresponding to line IV in Figure 3. cas: cervical air sac; cb: coracoid bone; cl: clavicle; clas: clavicular air sac; dcas: intrathoracic diverticula of clavicular air sac; H: humerus; M. longus colli dorsalis; mlv: M. longus colli ventralis; mmb: major metacarpal bone; mimb: minor metacarpal bone; o: oesophagus; pad: phalanx of alular digit; pm: pectoral muscle; ra: radius; rjv: right jugular vein; sc: spinal cord; Scp: scapulla; scm: supracoracoid muscle; tc: trachea; ul: ulna; v: vertebrae.

**Figure 8:**
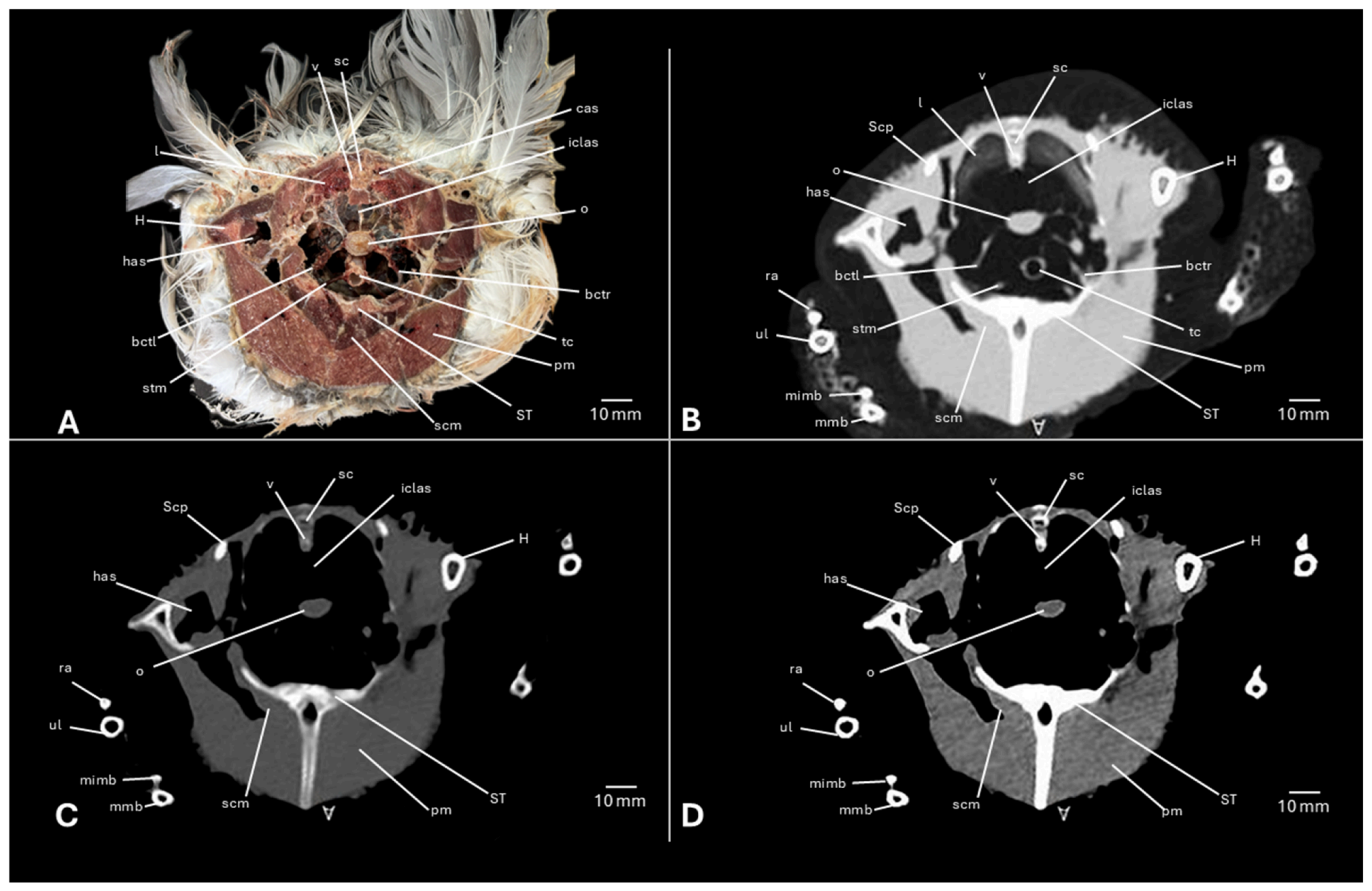
Transverse cross-sectional image (**A**), and pulmonary (**B**), bone (**C**) and soft tissue (**D**) CT images of the coelomic cavity of *the Larus michahellis* corresponding to line V in Figure 3. bctl: left brachiocephalic trunk; bctr: right brachiocephalic trunk; H: humerus; has: humeral air sac; iclas: interclavicular air sac; l: lung; mmb: major metacarpal bone; mimb: minor metacarpal bone; mlv: M. longus colli ventralis; ra: radius; sc: spinal cord; scm: supracoracoid muscle; Scp: scapulla; ST: sternum; stm: sternotracheal muscle; tc: trachea; o: oesophagus; pm: pectoral muscle; ul: ulna; v: vertebrae.

**Figure 9:**
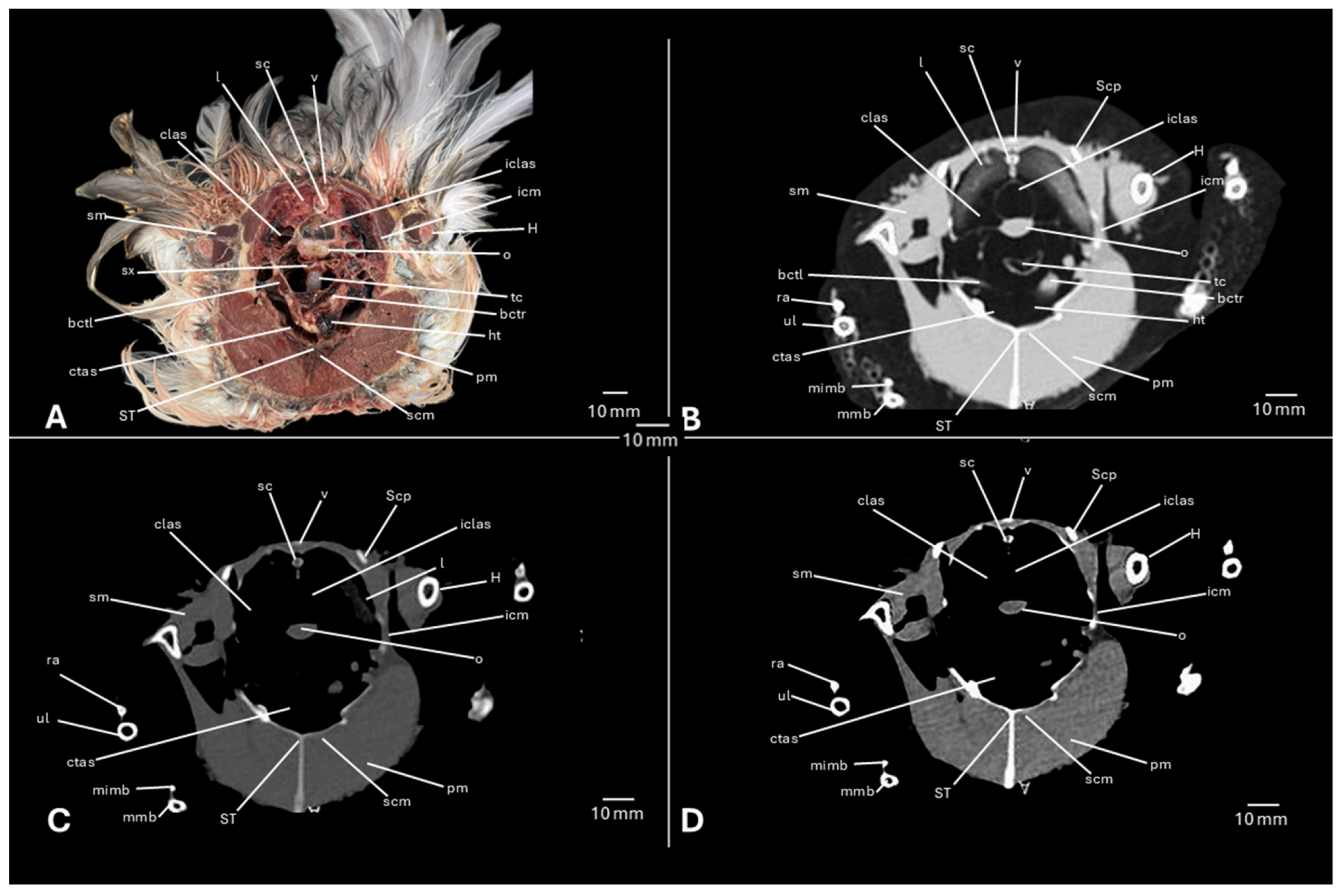
Transverse cross-sectional image (**A**), and pulmonary (**B**), bone (**C**) and soft tissue (**D**) CT images of the coelomic cavity of the *Larus michahellis* corresponding to line VI in Figure 3. bctl: left brachiocephalic trunk; bctr: right brachiocephalic trunk; clas: clavicular air sac; ctas: cranial thoracic air sac; H: humerus; ht: heart; iclas: interclavicular air sac; icm: intercostal muscle; l: lung; mmb: major metacarpal bone; mimb: minor metacarpal bone; o: oesophagus; pm: pectoral muscle; ra: radius; sc: spinal cord; scm: supracoracoid muscle; Scp: scapulla; sm: scapulohumeral caudal muscle; ST: sternum; sx: syrinx; tc: trachea; ul: ulna; v: vertebrae.

**Figure 10:**
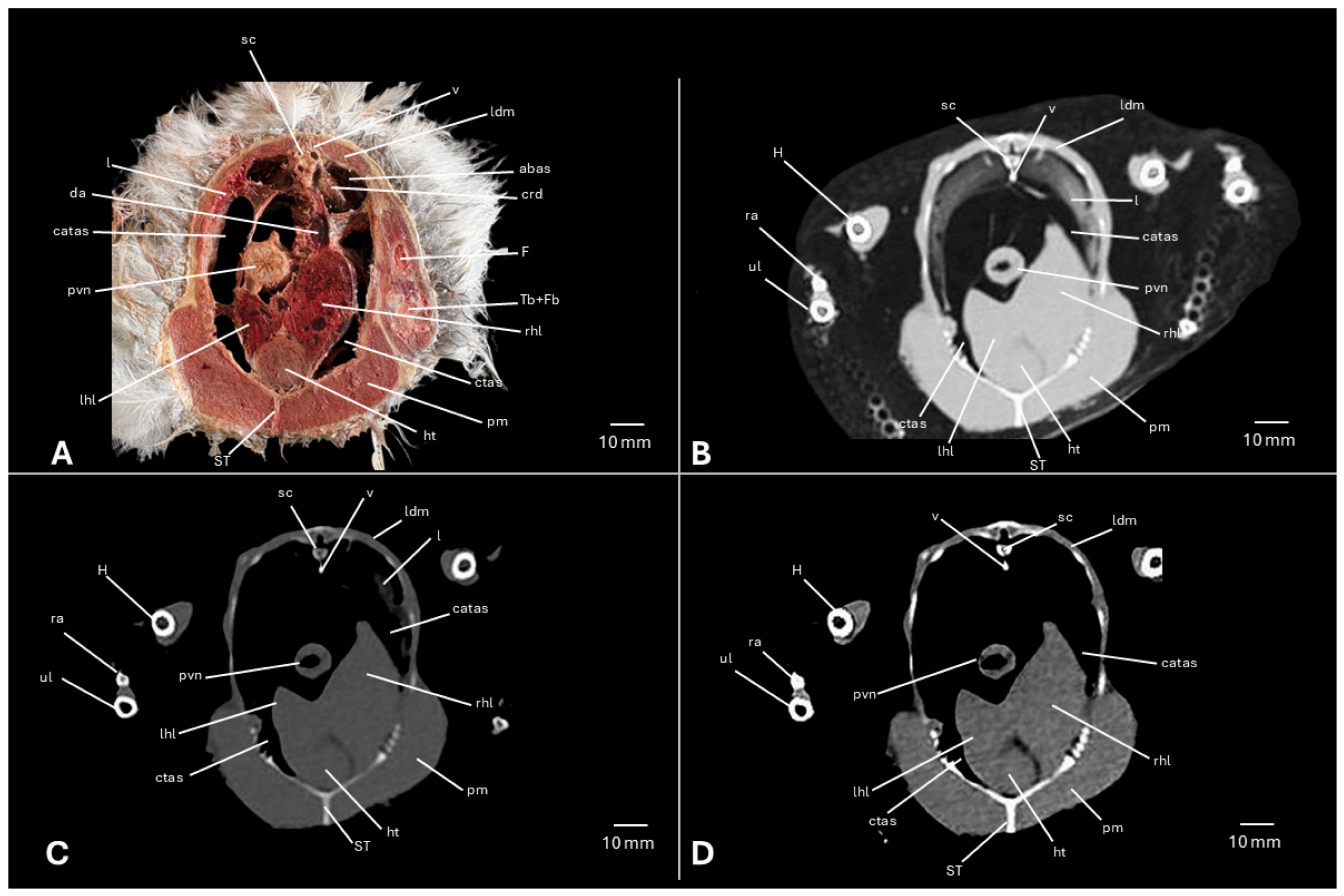
Transverse cross-sectional image (**A**), and pulmonary (**B**), bone (**C**) and soft tissue (**D**) CT images of the coelomic cavity of the *Larus michahellis* corresponding to line VII in Figure 3. abas: abdominal air sac; catas: caudal thoracic air sac; ctas: cranial thoracic air sac; crd: cranial renal division; da: descending aorta; F: femur; H: humerus; ht: heart; l: lung; ldm: *M. latissimus dorsi*; lhl: left hepatic lobe; pm: pectoral muscle; pvn: proventriculus; ra: radius; rhl: right hepatic lobe; sc: spinal cord; ST: sternum; Tb + Fb: tibiotarsus + fibula; ul: ulna; v: vertebrae.

**Figure 11:**
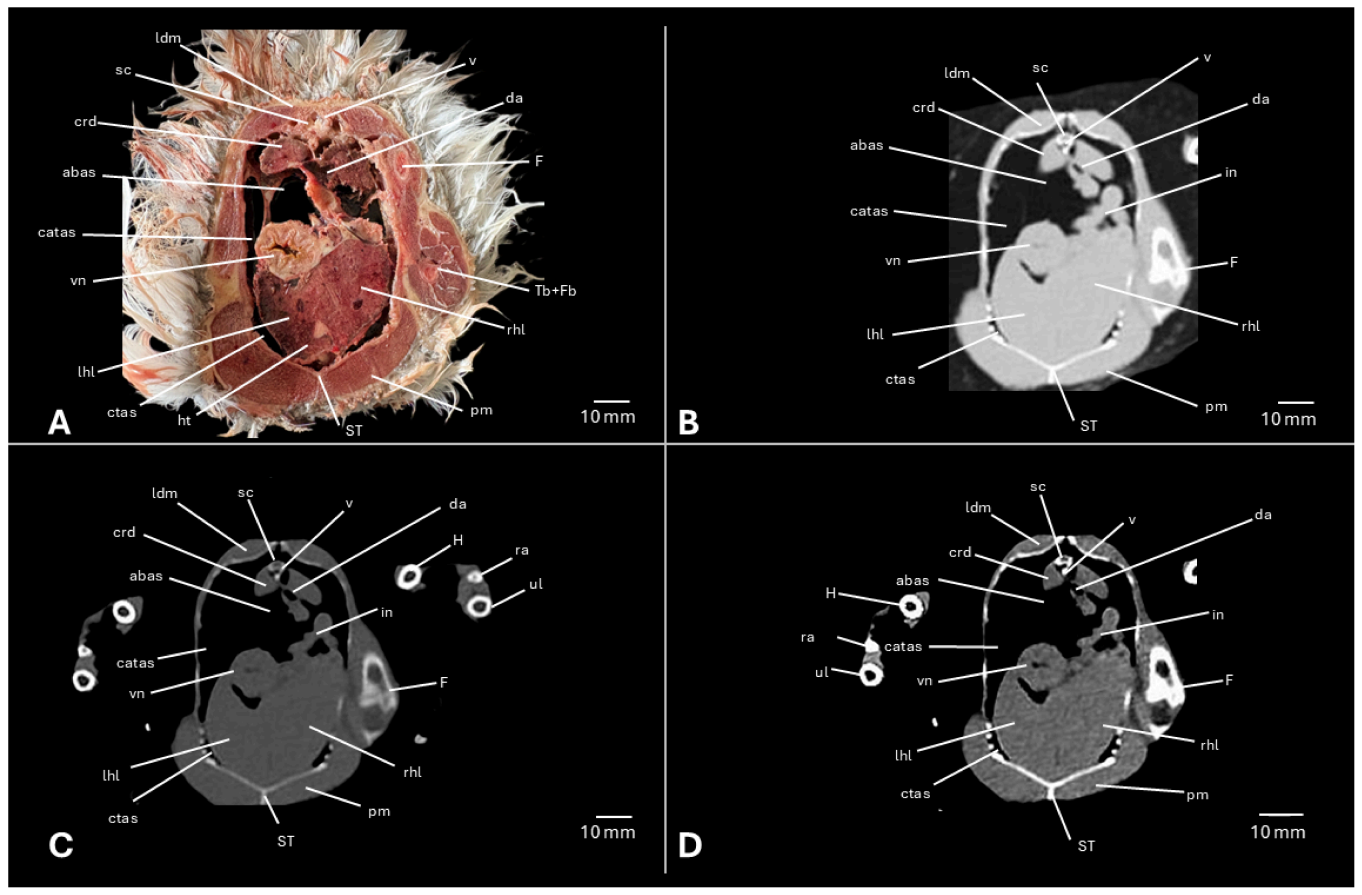
Transverse cross-sectional image (**A**), and pulmonary (**B**), bone (**C**) and soft tissue (**D**) CT images of the coelomic cavity of the *Larus michahellis* corresponding to line VIII in Figure 3. abas: abdominal air sac; catas: caudal thoracic air sac; ctas: cranial thoracic air sac; crd: cranial renal division; da: descending aorta; F: femur; H: humerus; ht: heart; in: intestines; ldm: *M. latissimus dorsi*; lhl: left hepatic lobe; pm: pectoral muscle; ra: radius; rhl: right hepatic lobe; sc: spinal cord; ST: sternum; Tb + Fb: tibiotarsus + fibula; ul: ulna; vn: ventriculus; v: vertebrae.

**Figure 12:**
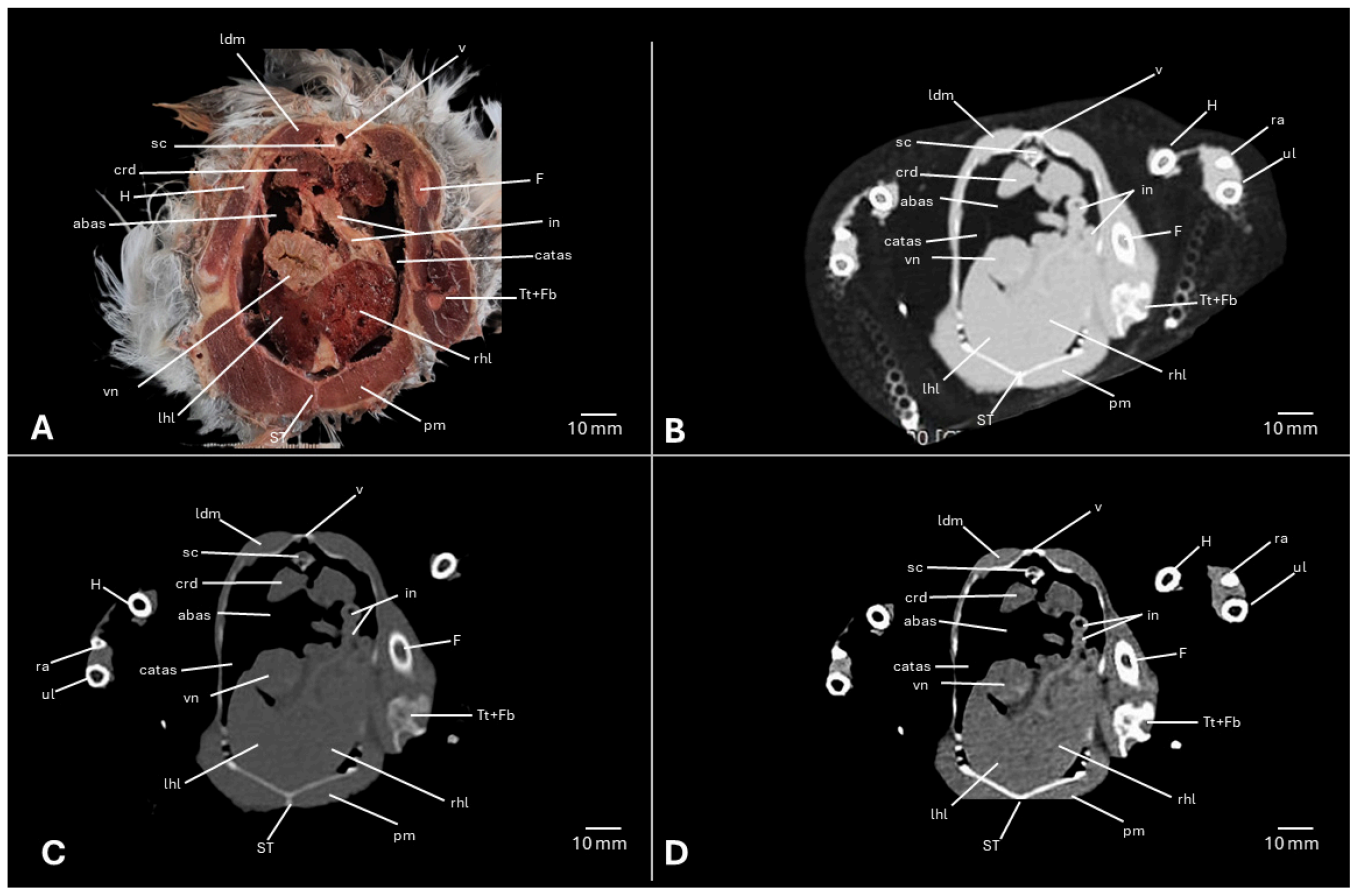
Transverse cross-sectional image (**A**), and pulmonary (**B**), bone (**C**) and soft tissue (**D**) CT images of the coelomic cavity of the *Larus michahellis* corresponding to line IX in Figure 3. abas: abdominal air sac; catas: caudal thoracic air sac; crd: cranial renal division; F: femur; H: humerus; in: intestines; ldm: M. latissimus dorsi; lhl: left hepatic lobe; pm: pectoral muscle; ra: radius; rhl: right hepatic lobe; sc: spinal cord; ST: sternum; Tt + Fb: tibiotarsus and fibula; ul: ulna; vn: ventriculus; v: vertebrae.

**Figure 13:**
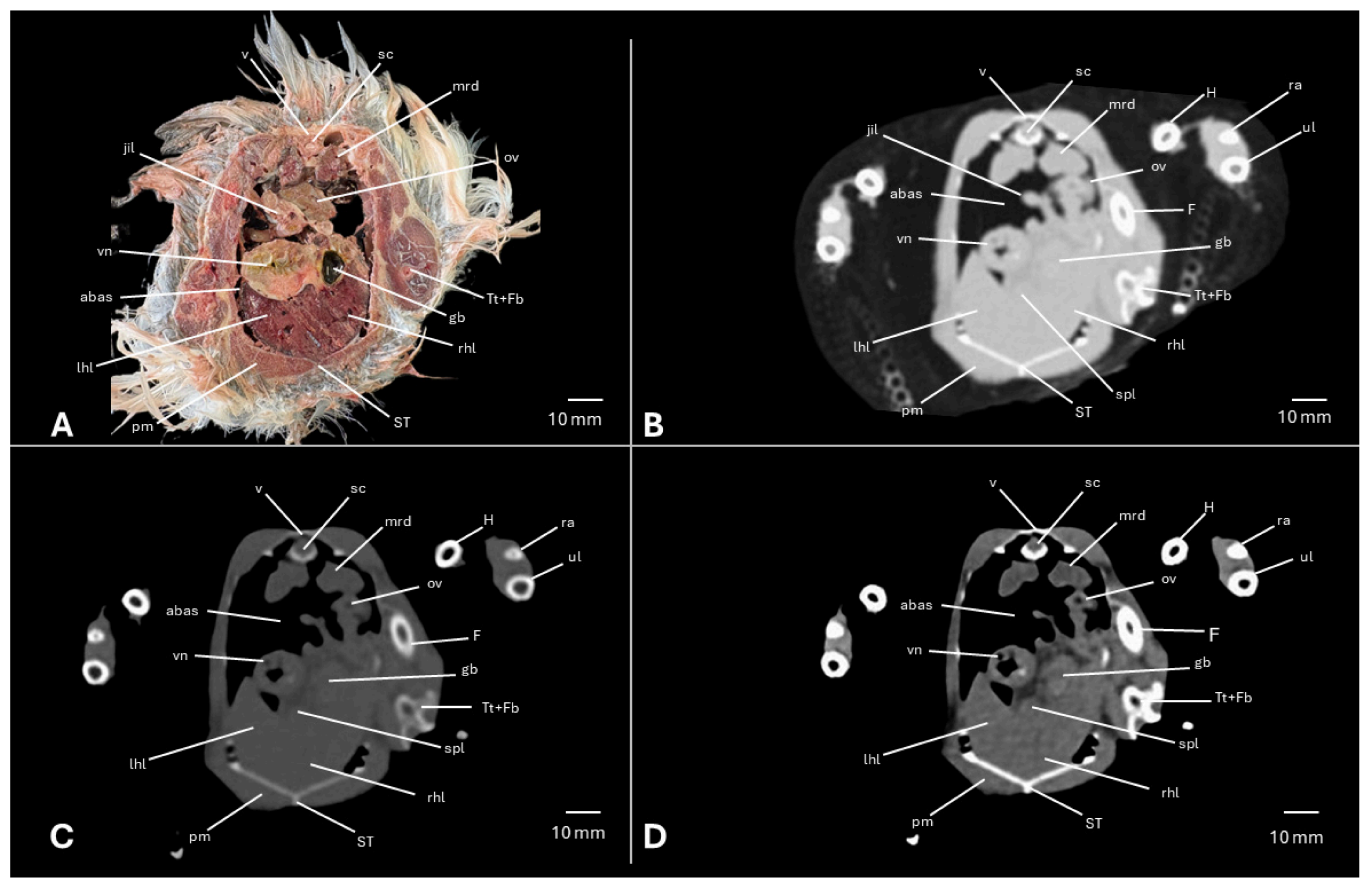
Transverse cross-sectional image (**A**), and pulmonary (**B**), bone (**C**) and soft tissue (**D**) CT images of the coelomic cavity of the *Larus michahellis* corresponding to line X in Figure 3. abas: abdominal air sac; F: femur gb: gallbladder; H: humerus; jil: jejunoileal loops; lhl: left hepatic lobe; mdr: middle renal division; ov: left ovary; pm: pectoral muscle; rhl: right hepatic lobe; ra: radius; sc: spinal cord; spl: spleen; ST: sternum; Tt + Fb: tibiotarsus and fibula; ul: ulna; v: vertebrae; vn: ventriculus.

**Figure 14:**
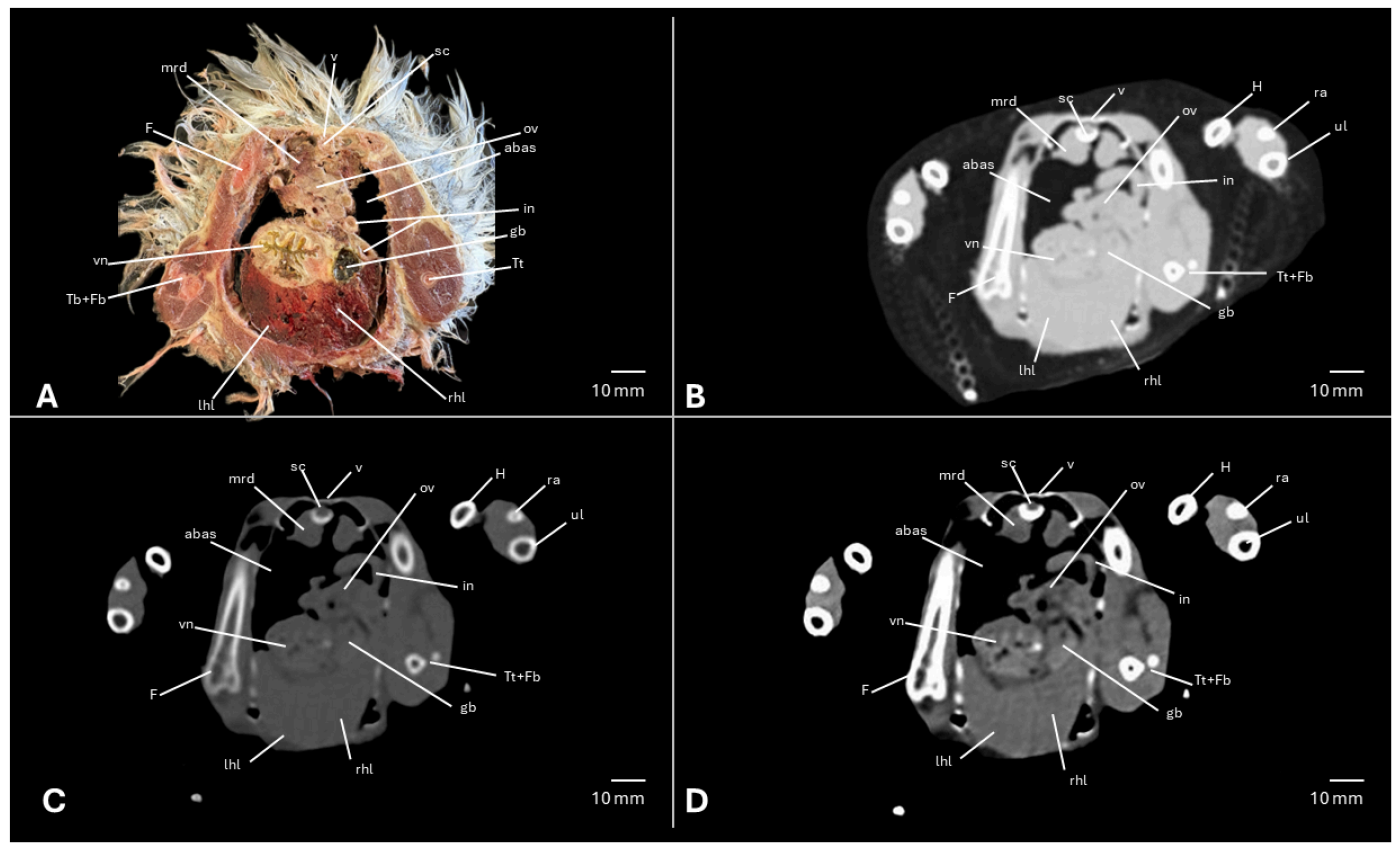
Transverse cross-sectional image (**A**), and pulmonary (**B**), bone (**C**) and soft tissue (**D**) CT images of the coelomic cavity of the *Larus michahellis* corresponding to line XI in Figure 3. abas: abdominal air sac; F: femur; gb: gallbladder; H: humerus; in: intestines; lhl: left hepatic lobe; mrd: median renal division; ov: left ovary; ra: radius; rhl: right hepatic lobe; sc: spinal cord; Tt + Fb: tibiotarsus and fibula; ul: ulna; v: vertebrae; vn: ventriculus.

**Figure 15:**
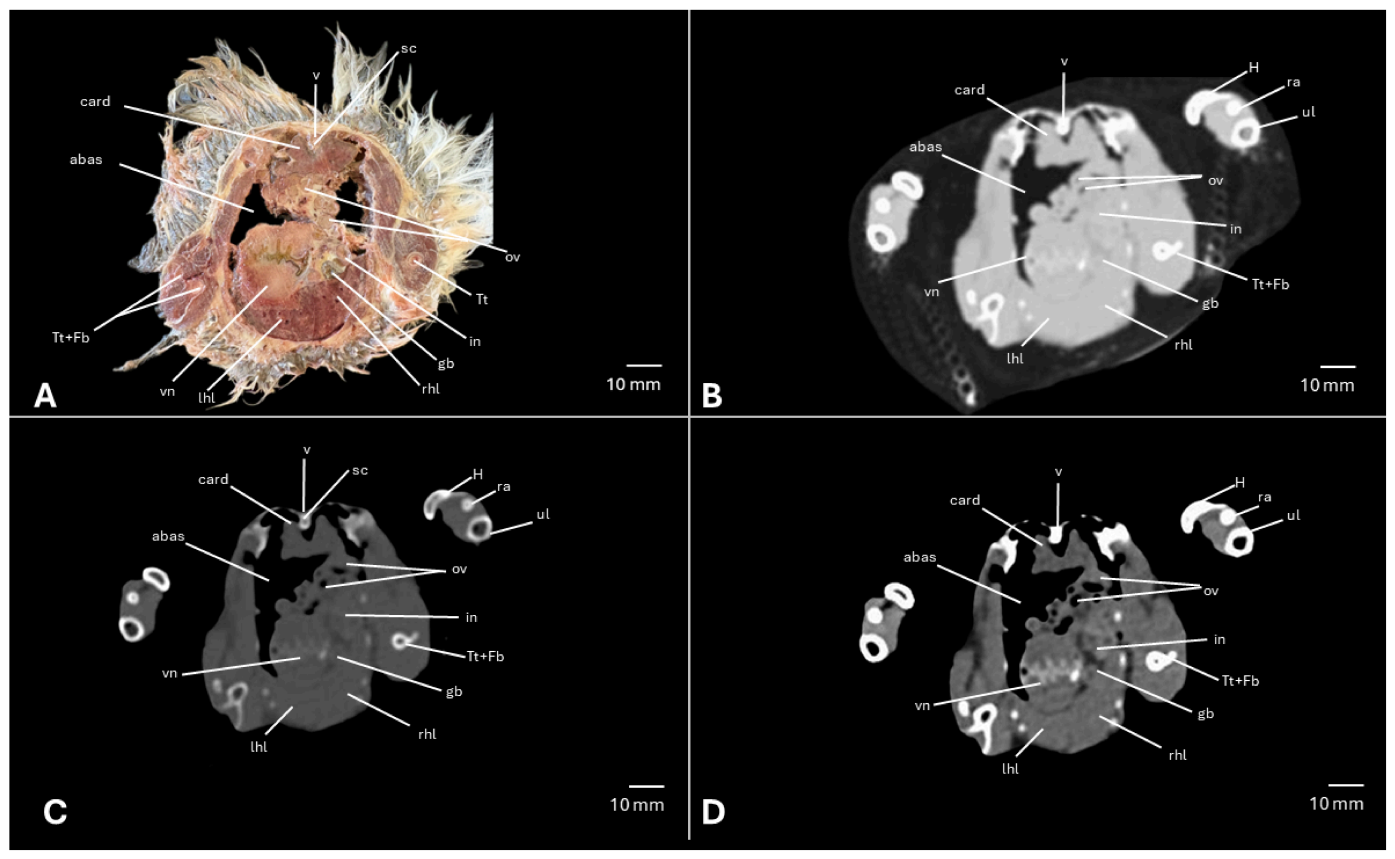
Transverse cross-sectional image (**A**), and pulmonary (**B**), bone (**C**) and soft tissue (**D**) CT images of the coelomic cavity of the *Larus michahellis* corresponding to line XII in Figure 3. abas: abdominal air sac; card: caudal renal division; H: humerus; gb: gallbladder; in: intestine; lhl: left hepatic lobe; ov: left ovary; rhl: right hepatic lobe; ra: radius; sc: spinal cord; Tt + Fb: tibiotarsus and fibula; ul: ulna; v: vertebrae; vn: ventriculus.

**Figure 16:**
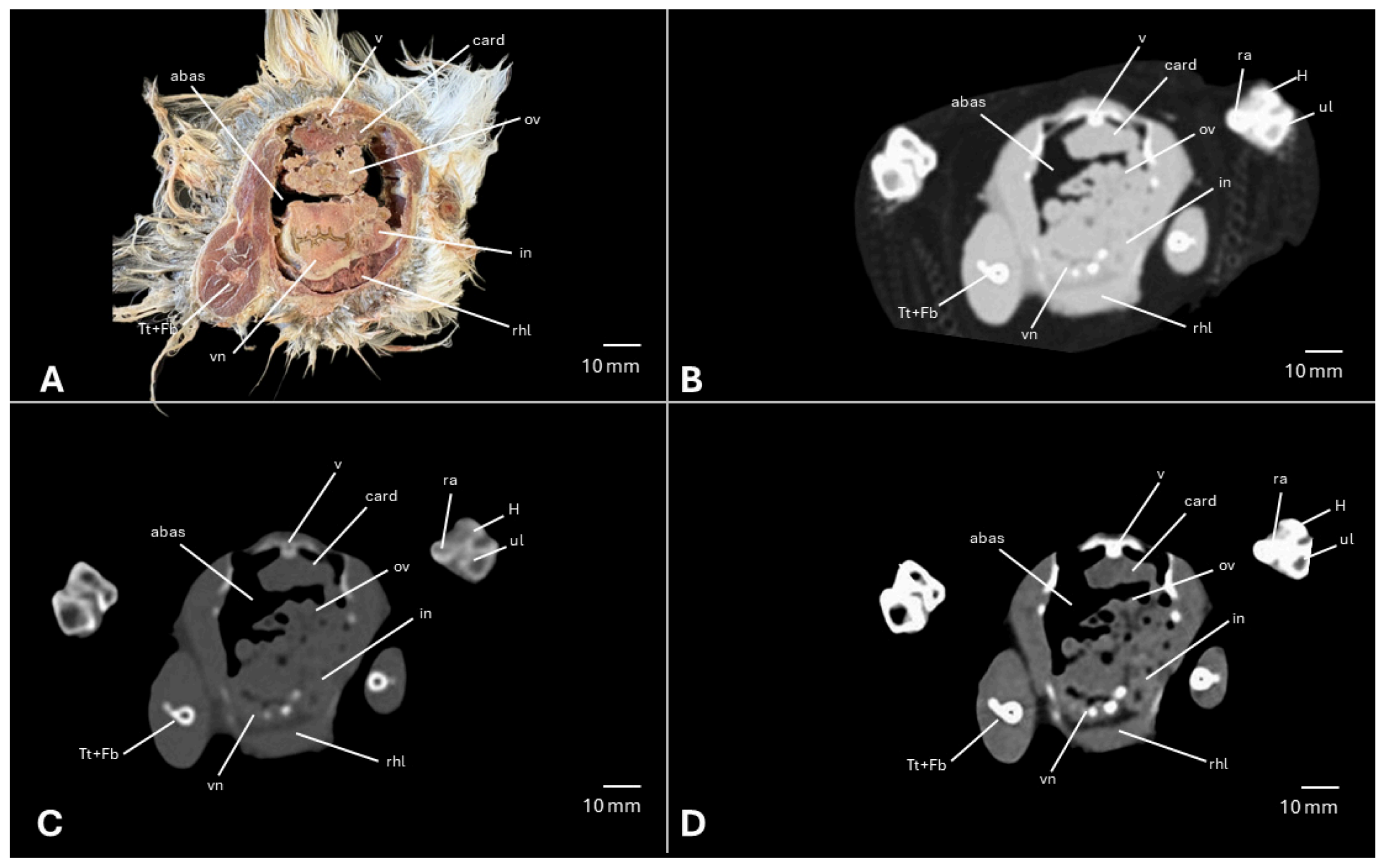
Transverse cross-sectional image (**A**), and pulmonary (**B**), bone (**C**) and soft tissue (**D**) CT images of the coelomic cavity of the *Larus michahellis* corresponding to line XIII in Figure 3. abas: abdominal air sac; card: caudal renal division; H: humerus; in: intestines; ov: left ovary; ra: radius; rhl: right hepatic lobe; Tt + Fb: tibiotarsus and fibula; ul: ulna; v: vertebrae; vn: ventriculus.

**Figure 17:**
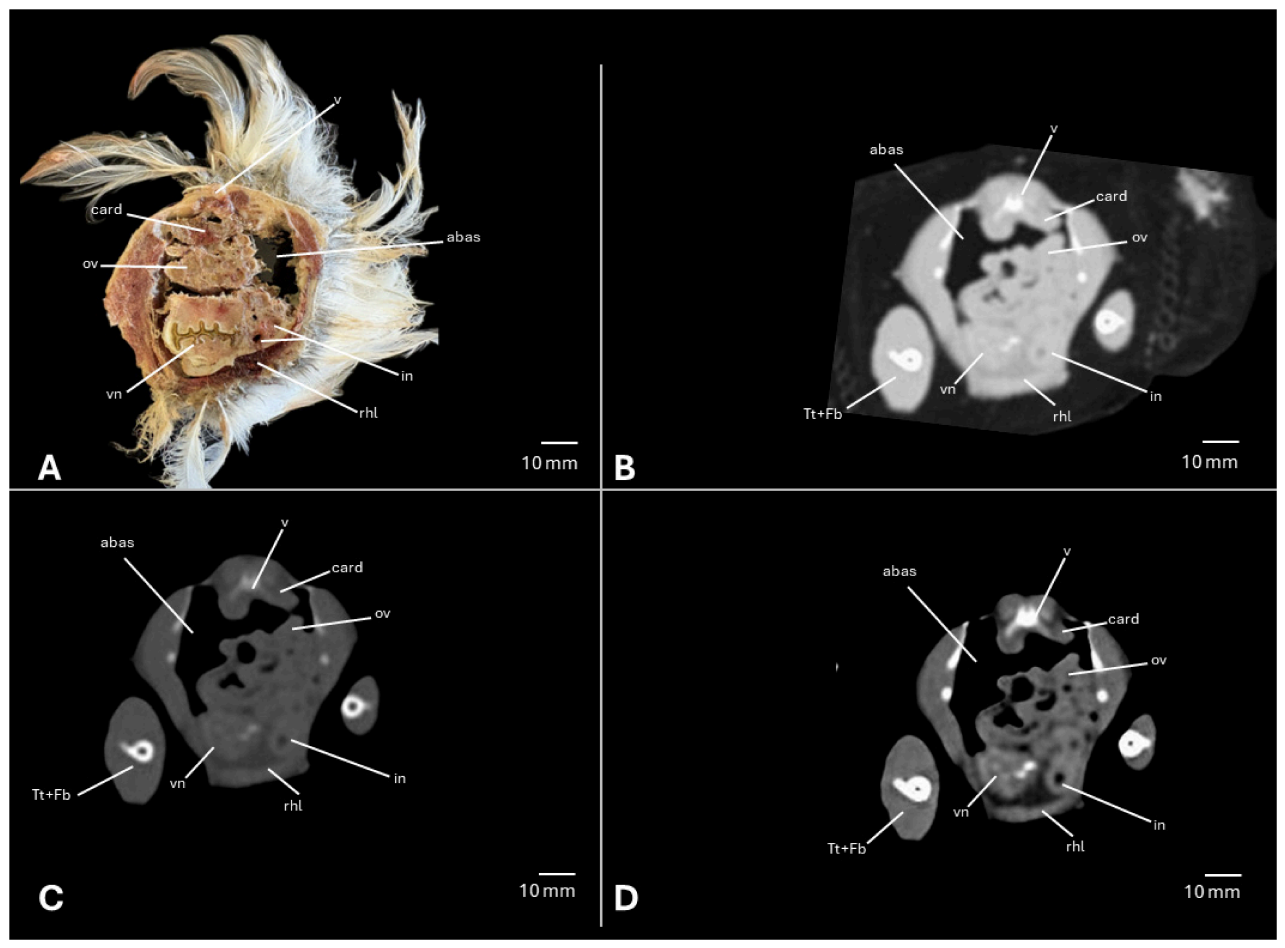
Transverse cross-sectional image (**A**), and pulmonary (**B**), bone (**C**) and soft tissue (**D**) CT images of the coelomic cavity of the *Larus michahellis* corresponding to line XIV in Figure 3. abas: abdominal air; card: caudal renal division; in: intestines; ov: left ovary; rhl: right hepatic lobe; Tt + Fb: tibiotarsus and fibula v: vertebrae; vn: ventriculus.

**Figure 18:**
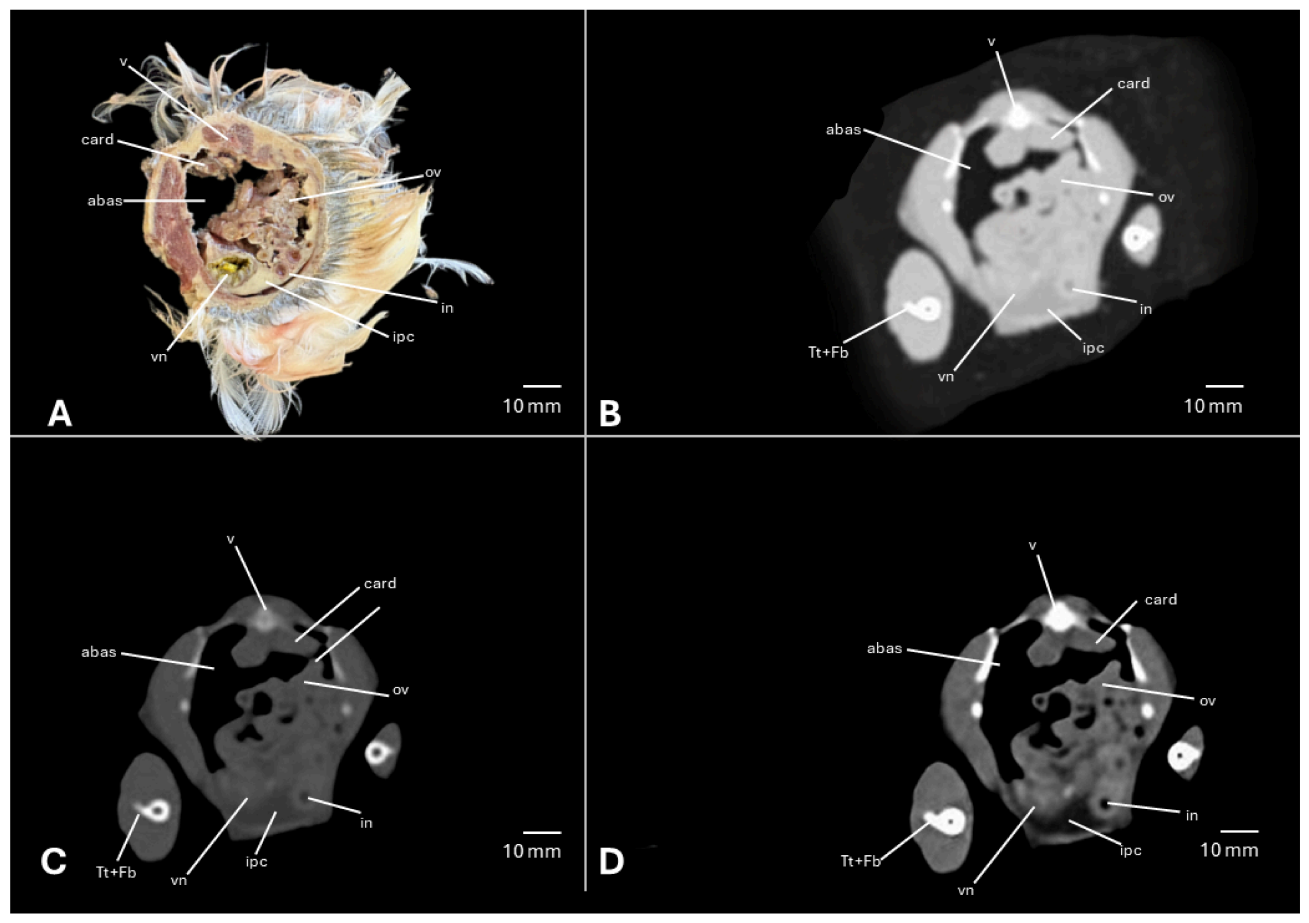
Transverse cross-sectional image (**A**), and pulmonary (**B**), bone (**C**) and soft tissue (**D**) CT images of the coelomic cavity of the *Larus michahellis* corresponding to line XV in Figure 3. abas: abdominal air sac; card: caudal renal division; in: intestine; ipc: intestinal peritoneal cavity with fat; ov: left ovary; Tt + Fb: tibiotarsus and fibula; vn: ventriculus; v: vertebrae.

**Figure 19:**
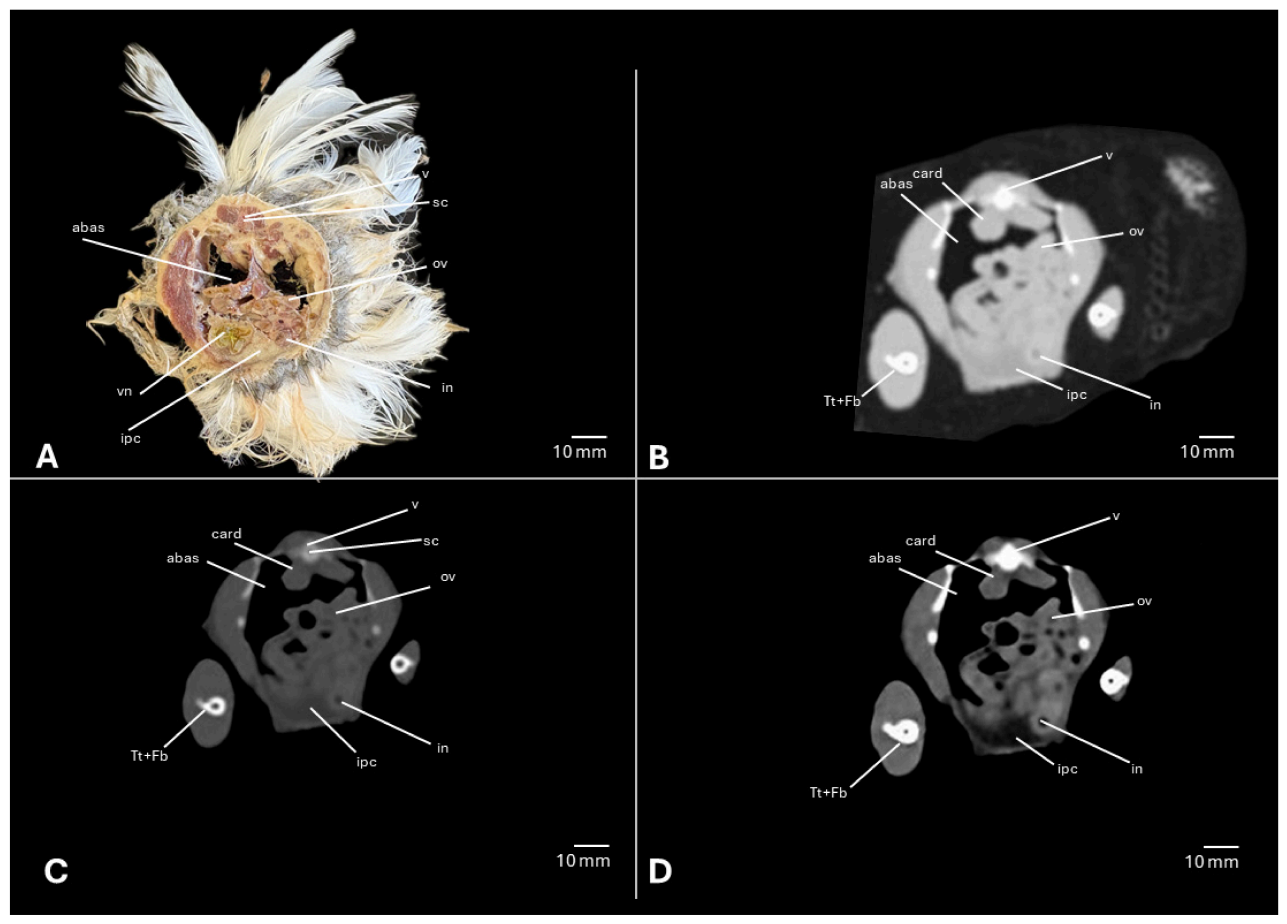
Transverse cross-sectional image (**A**), and pulmonary (**B**), bone (**C**) and soft tissue (**D**) CT images of the coelomic cavity of the *Larus michahellis* corresponding to line XVI in Figure 3. abas: abdominal air sac; card: caudal renal division; in: intestines; ipc: intestinal peritoneal cavity with fat; ov: left ovary; sc: spinal cord; Tt + Fb: tibiotarsus and fibula; vn: ventriculus; v: vertebrae.

**Figure 20:**
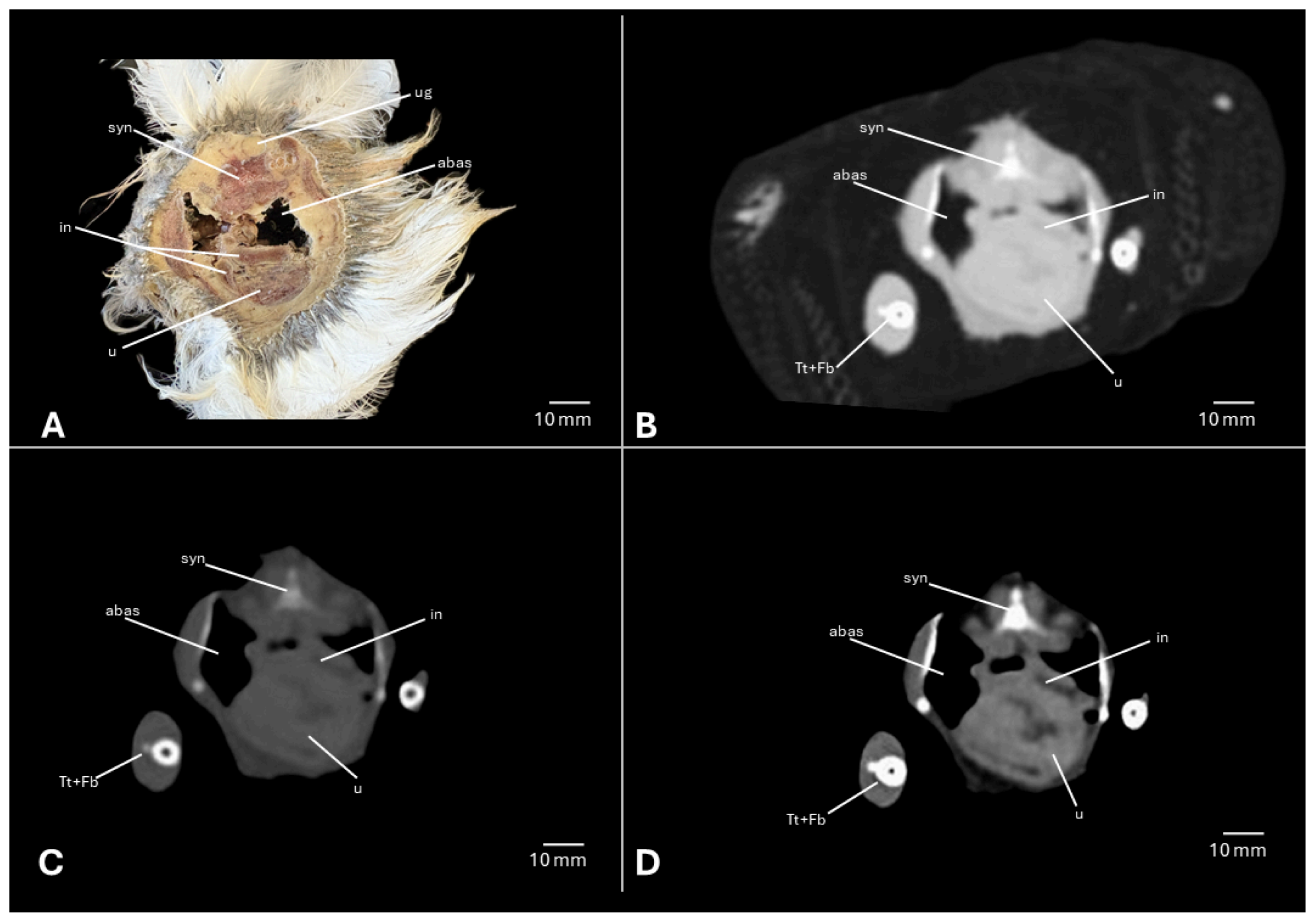
Transverse cross-sectional image (**A**), and pulmonary (**B**), bone (**C**) and soft tissue (**D**) CT images of the coelomic cavity of the *Larus michahellis* corresponding to line XVII in Figure 3. abas: abdominal air sac; in: intestines; syn: synsacrum; Tt + Fb: tibiotarsus and fibula; u: uterus; ug: uropygial gland.

**Figure 21:**
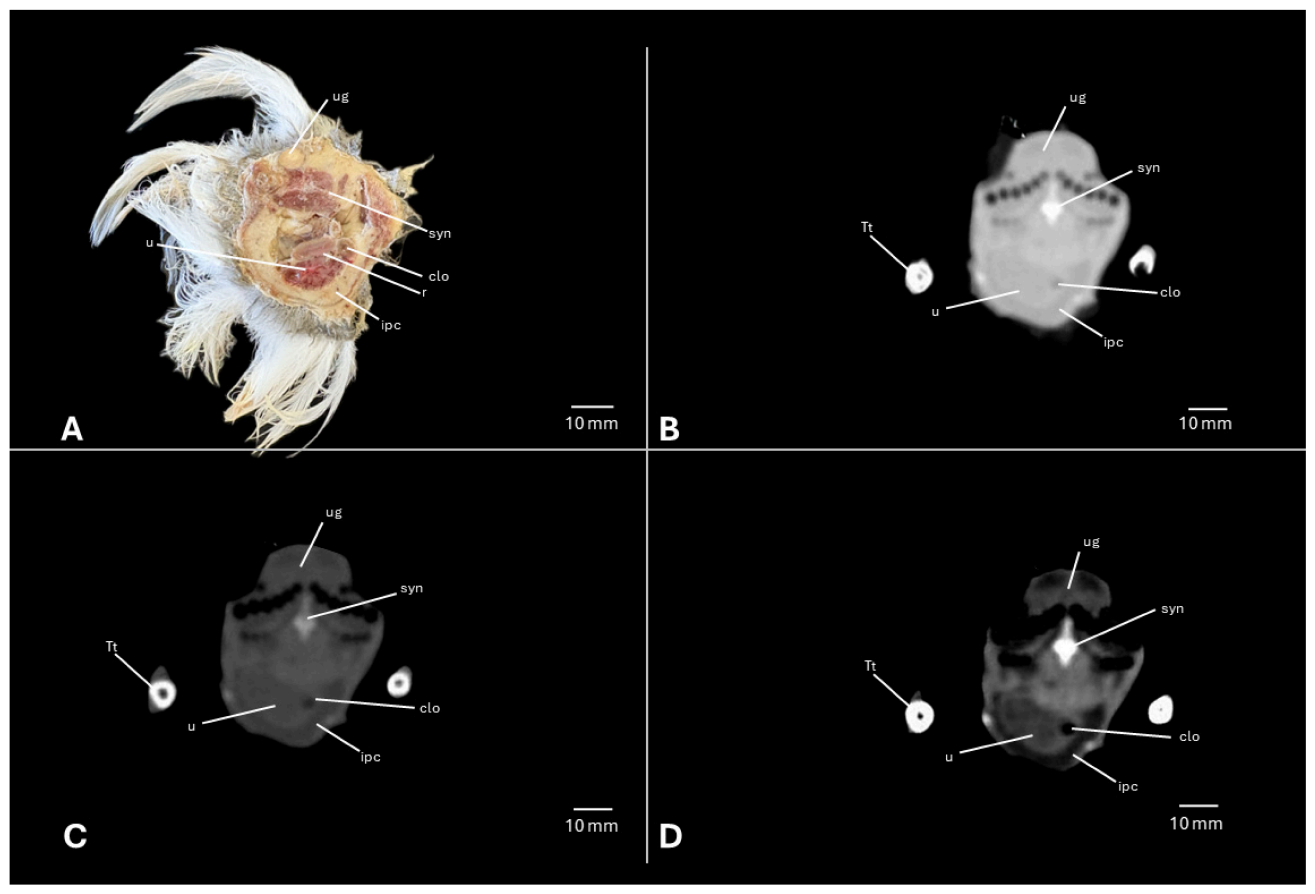
Transverse cross-sectional image (**A**), and pulmonary (**B**), bone (**C**) and soft tissue (**D**) CT images of the coelomic cavity of the *Larus michahellis* corresponding to line XVIII in Figure 3. clo: cloaca; ipc: intestinal peritoneal cavity with fat; r: rectum; syn: synsacrum; Tt: tibiotarsus; u: uterus; ug: uropygial gland.

**Figure 22:**
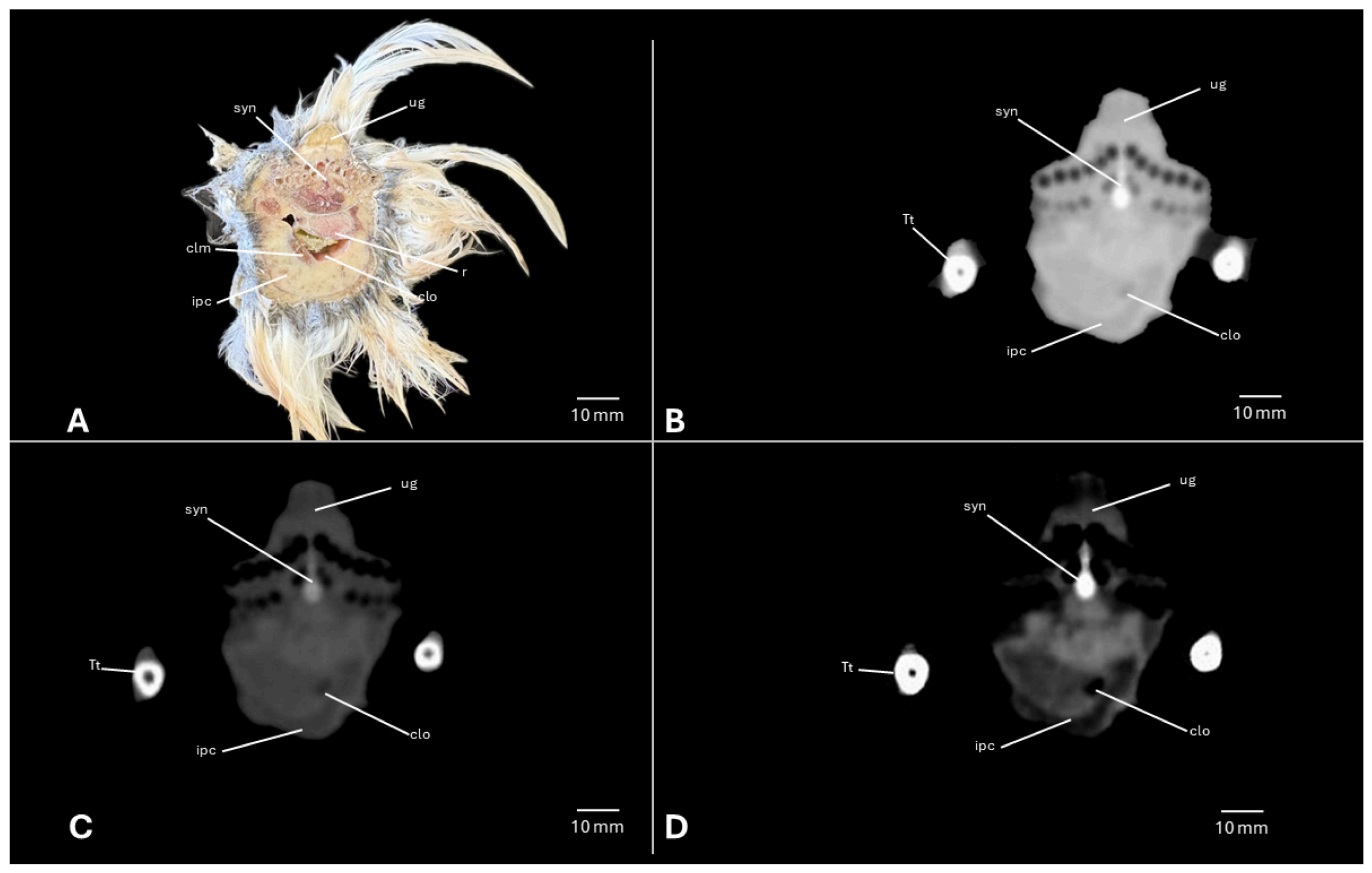
Transverse cross-sectional image (**A**), and pulmonary (**B**), bone (**C**) and soft tissue (**D**) CT images of the coelomic cavity of the *Larus michahellis* corresponding to line XIX in Figure 3. clo: cloaca; clm: cloacal muscles; ipc: intestinal peritoneal cavity with fat; r: rectum (terminal part); syn: synsacrum; Tt: tibiotarsus; ug: uropygial gland.

**Figure 23:**
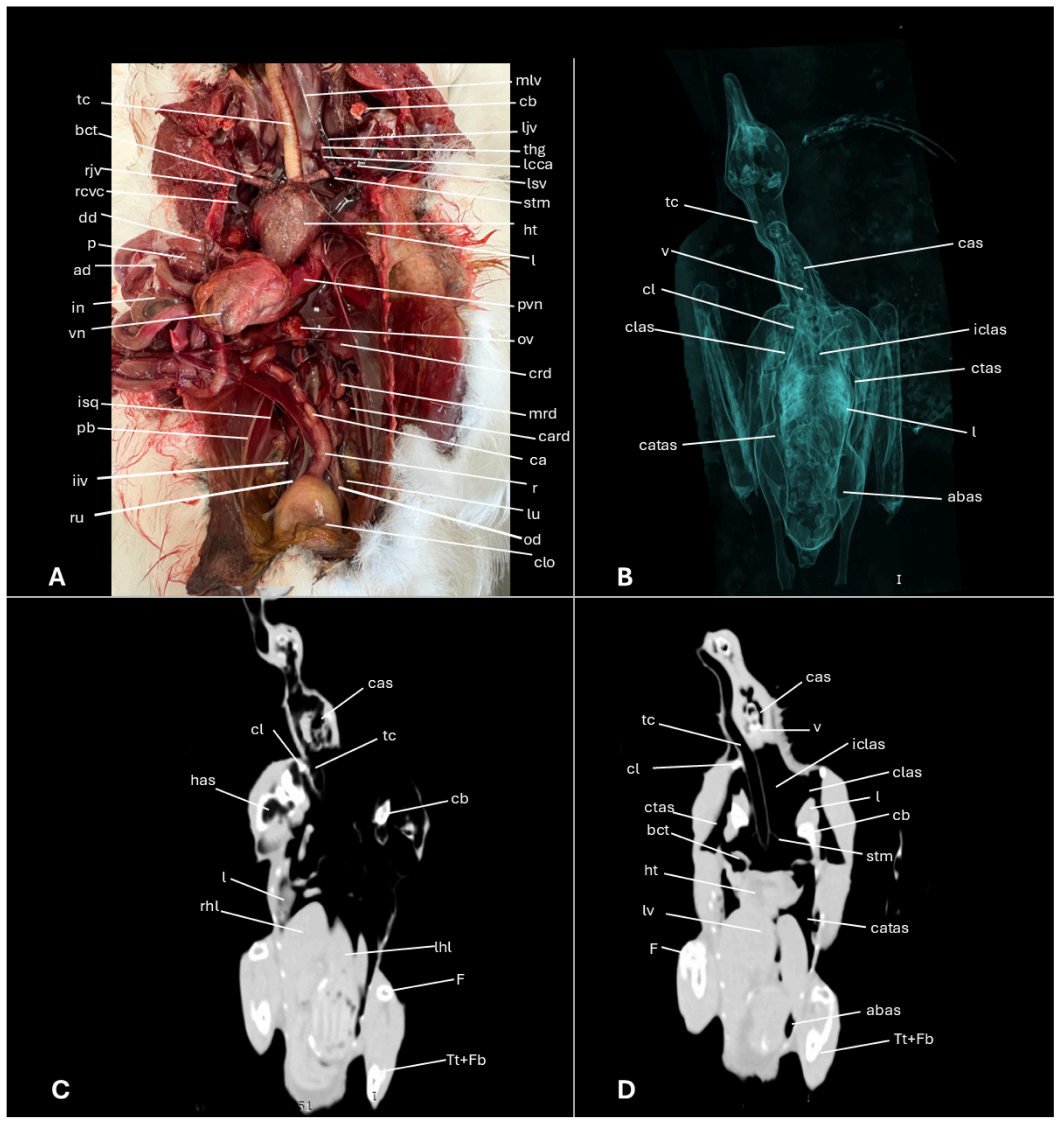
Anatomical dissection illustrates the coelomic cavity of the *Larus michahellis* after removing the liver, gallbladder and spleen (**A**), OsiriX MIP reconstruction image (**B**) and dorsal MPR images in pulmonary CT window of the coelomic cavity at level of liver (**C**) and heart (**D**). abas: abdominal air sac; ad: ascending duodenum; bct: brachiocephalic trunk; ca: caecums; card: caudal renal division; cas: cervical air sac; catas: caudal thoracic air sac; ctas: cranial thoracic air sac; cb: coracoid bone; cl: clavicle; clas: clavicular air sac; clo: cloaca; crd: cranial renal division; dd: descending duodenum; F: femur; has: humeral air sac; ht: heart; iclas: interclavicular air sac; iiv: internal iliac vessels; in: intestine; isq: ischium; l: lung; lcca: left common carotid artery; ljv: left jugular vein; lhl: left hepatic lobe; lsv: left subclavian vein; lu: left ureter; lv: liver; mlv: *M. longus colli ventralis*; mrd: middle renal division; od: left oviduct; ov: left ovary; p: pancreas; pb: pubis; pvn: proventriculus; r: rectum; rcvc: right cranial vena cava; rhl: right hepatic lobe; rjv: right jugular vein; ru: right ureter; stm: sternotracheal muscle; tc: trachea; thg: thyroid gland; Tt + Fb: tibiotarsus and fibula; v: vertebrae; vn: ventriculus.

A detailed set of figures (Figure 1, Figure 2, Figure 3, Figure 4, Figure 5, Figure 6, Figure 7, Figure 8, Figure 9, Figure 10, Figure 11, Figure 12, Figure 13, Figure 14, Figure 15, Figure 16, Figure 17, Figure 18, Figure 19, Figure 20, Figure 21, Figure 22 and Figure 23) illustrates the anatomical organization of the coelomic cavity in gulls, constituting a structured atlas based on combined dissection, anatomical cross-sections and imaging approaches. These illustrations integrate multiple dissection views, providing adequate anatomical references for the main structures within the cavity. In particular, Figure 1 and Figure 2 compile different dissection perspectives to present an overall view of the principal components and their spatial relationships. Importantly, this atlas-based approach facilitates direct correlation with CT images, enhancing the interpretation of imaging findings and supporting a more accurate understanding of three-dimensional anatomical organization.

### 3.1. Anatomical Dissections and Cross-Sections

Anatomical dissections (Figure 1A–C, Figure 2A–C and Figure 23A) and transverse sections (Figure 4A, Figure 5A, Figure 6A, Figure 7A, Figure 8A, Figure 9A, Figure 10A, Figure 11A, Figure 12A, Figure 13A, Figure 14A, Figure 15A, Figure 16A, Figure 17A, Figure 18A, Figure 19A, Figure 20A, Figure 21A and Figure 22A) of the coelomic cavity in gulls are shown. These images allow the identification of different components of the respiratory, cardiovascular, digestive, female genital and urinary systems within this cavity. The observations reveal the anatomical organization of the coelomic cavity, highlighting the spatial relationships among the contained organs.

Within this cavity, the thyroid glands were identified as round structures located at the base of the neck, positioned bilaterally to the trachea (Figure 1A,B and Figure 23A). Each gland was closely associated with the common carotid arteries. The parathyroid glands were also observed at the caudal pole of the thyroid glands. The trachea followed a median course into the coelomic cavity, where it bifurcated into the right and left primary bronchi, a feature evident in the transverse sections (Figure 1A,B, Figure 4A, Figure 5A, Figure 6A, Figure 7A, Figure 8A, Figure 9A and Figure 23A). The syrinx was identified in selected anatomical preparations (Figure 1B and Figure 9A). Additional structures associated with the trachea included the cervical portion of the oesophagus, the *longissimus colli* muscle, and the sternotracheal muscles (Figure 1B, Figure 4A, Figure 5A, Figure 6A, Figure 7A, Figure 8A and Figure 9A).

Both lungs occupied a craniodorsal position, adjacent to the ribs (Figure 1A,C, Figure 2A,B, Figure 8A, Figure 9A, Figure 10A and Figure 23A). The combined analysis of dissections and cross-sections also allowed the identification of the wall of the cervical, clavicular, interclavicular, cranial and caudal thoracic, and abdominal air sacs (Figure 1A,C, Figure 2A,B, Figure 5A, Figure 6A, Figure 7A, Figure 8A, Figure 9A, Figure 10A, Figure 11A, Figure 12A, Figure 13A, Figure 14A, Figure 15A, Figure 16A, Figure 17A, Figure 18A, Figure 19A and Figure 20A). The heart appeared as an ovoid structure with a pointed apex, and positioned cranial to the liver (Figure 1A,B, Figure 2A,B, Figure 9A, Figure 10A, Figure 11A, Figure 12A and Figure 23A). Major vascular structures, including the brachiocephalic trunks, the left and right subclavian veins, the left and right jugular veins, and the left and right cranial venae cavae, were also recognized (Figure 1A,B, Figure 2A, Figure 4A, Figure 5A, Figure 6A, Figure 7A, Figure 8A, Figure 9A and Figure 23A). Caudal to the heart, the liver was divided into left and right hepatic lobes, with the gallbladder located on the visceral surface of the right hepatic lobe (Figure 1A,C, Figure 2B,C, Figure 10A, Figure 11A, Figure 12A, Figure 13A, Figure 14A, Figure 15A, Figure 16A and Figure 17A). Closely associated with the visceral hepatic surface, the spleen appeared as an elongated and narrow structure (Figure 2C).

More caudally, several components of the digestive tract could be distinguished. The proventriculus was located on the left side of the coelomic cavity at the junction between the oesophagus and the ventriculus (Figure 2A, Figure 10A and Figure 23A). The ventriculus was closely associated with the visceral surface of the left hepatic lobe and exhibited a markedly thick muscular wall, making it readily identifiable in these cross-sections (Figure 1A,C, Figure 2A,B, Figure 11A, Figure 12A, Figure 13A, Figure 14A, Figure 15A, Figure 16A, Figure 17A, Figure 18A, Figure 19A and Figure 23A). The duodenum was more clearly appreciated in the dissected views caudal to the liver (Figure 1A,C, Figure 2A,B and Figure 23A), where it formed a U-shaped *ansa duodeni* composed of descending (*pars descendens*) and ascending (*pars ascendens*) segments. Between these segments, the pancreas was identified within the mesoduodenum and showed a characteristic red-dish-brown appearance (Figure 1A,C, Figure 2B and Figure 23A). Jejunoileal loops occupied the mid and right caudal regions of the coelomic cavity. Regarding the large intestine, two small and poorly developed ceca were observed together with the rectum, which extended caudally towards the cloaca (Figure 2A and Figure 23A). The cloaca appeared as a large caudomedian cavity located ventral to the terminal rectum and receiving the distal portion of the left oviduct. This structure was surrounded by well-developed cloacal musculature, which facilitates substantial enlargement during physiological processes such as oviposition, copulation, and defecation (Figure 1A,C, Figure 2A, Figure 21A, Figure 22A and Figure 23A).

The urinary system was clearly distinguished following liver removal in the dissected specimens. Transverse sections enabled the recognition of kidneys positioned laterally to the vertebral column, dorsally embedded within the synsacral fossae and closely associated with the abdominal air sacs. Three renal divisions, namely cranial, middle, and caudal divisions, were differentiated (Figure 2A, Figure 10A, Figure 11A, Figure 12A, Figure 13A, Figure 14A, Figure 15A, Figure 16A, Figure 17A, Figure 18A and Figure 23A).

Finally, the dissected and transverse images facilitated the identification of the female reproductive tract. The left ovary, containing multiple follicles, was located adjacent to the ventral aspect of the renal divisions (Figure 2A, Figure 13A, Figure 14A, Figure 15A, Figure 16A, Figure 17A, Figure 18A, Figure 19A and Figure 23A). In addition, the left oviduct occupied the left and caudal portion of the coelomic cavity within the intestinal peritoneal cavity and extended caudally towards the cloaca (Figure 2A and Figure 23A).

### 3.2. Computed Tomography Images

Computed tomography images corresponding to the dissections and anatomical transverse sections were selected for comparative evaluation (Figure 4B–D, Figure 22B–D and Figure 23C,D). Notably, CT images obtained with the pulmonary window setting (Figure 4B, Figure 5B, Figure 6B, Figure 7B, Figure 8B, Figure 9B, Figure 10B, Figure 11B, Figure 12B, Figure 13B, Figure 14B, Figure 15B, Figure 16B, Figure 17B, Figure 18B, Figure 19B, Figure 20B, Figure 21B, Figure 22B and Figure 23C,D) showed remarkable visualization of different bones, related muscles, and soft tissues. As a result, we differentiated between several skeletal formations, which included the clavicle, ulna, scapulla, thoracic vertebrae, sternum, humerus and femur. In addition, we examined a variety of muscles connected to these skeletal elements, such as the sternotracheal, scapulohumeralis, scapulohumeral caudal, longissimus colli, longissimus dorsi, pectoral and intercostal muscles (Figure 4B, Figure 5B, Figure 6B, Figure 7B, Figure 8B, Figure 9B, Figure 10B, Figure 11B, Figure 12B, Figure 13B and Figure 23D). More precisely, this window facilitated the identification of the trachea and the lung parenchyma (Figure 4B, Figure 5B, Figure 6B, Figure 7B, Figure 8B, Figure 9B, Figure 10B and Figure 23B,C,D). It also proved useful for identifying intrathoracic formations, including the heart and relevant vessels, such as the brachiocephalic trunks (Figure 6B, Figure 7B, Figure 8B, Figure 9B, Figure 10B and Figure 23D). Additionally, the walls of some air sacs, such as those of the cervical, clavicular, thoracic and abdominal air sacs, could be distinguished (Figure 4B, Figure 5B, Figure 6B, Figure 7B, Figure 8B, Figure 9B, Figure 10B, Figure 11B, Figure 12B, Figure 13B, Figure 14B, Figure 15B, Figure 16B, Figure 17B, Figure 18B, Figure 19B, Figure 20B and Figure 23B,C,D). Regarding the digestive components, this window clearly delineated the oesophagus, crop, hepatic lobes, glandular and muscular stomach components, various intestinal segments, and the cloaca (Figure 5B, Figure 6B, Figure 7B, Figure 8B, Figure 9B, Figure 10B, Figure 11B, Figure 12B, Figure 13B, Figure 14B, Figure 15B, Figure 16B, Figure 17B, Figure 18B, Figure 19B, Figure 20B, Figure 21B, Figure 22B and Figure 23C,D). In addition, this technology allowed structures from the genitourinary system to be clarified. The three cranial divisions were clearly distinguished following a dorsal position in the coelomic cavity (Figure 11B, Figure 12B, Figure 13B, Figure 14B, Figure 15B, Figure 16B, Figure 17B, Figure 18B and Figure 19B). Lateroventrally to these divisions, the left ovary was appreciable (Figure 13B, Figure 14B, Figure 15B, Figure 16B, Figure 17B, Figure 18B and Figure 19B).

CT images acquired using the bone and soft tissue window settings (Figure 4C,D and Figure 22C,D) demonstrated a remarkable distinction between bones and soft tissues. Interestingly, the different heart chambers and several vessels could be distinguished with these windows (Figure 6C,D, Figure 7C,D and Figure 10C,D). Moreover, essential digestive structures, such as the oesophagus, the hepatic lobes, different intestinal segments, as well as the glandular and muscular portions of the gull’s stomach, and the cloaca were also displayed (Figure 5C,D, Figure 6C,D, Figure 7C,D, Figure 8C,D, Figure 9C,D, Figure 10C,D, Figure 11C,D, Figure 12C,D, Figure 13C,D, Figure 14C,D, Figure 15C,D, Figure 16C,D, Figure 17C,D, Figure 18C,D, Figure 19C,D, Figure 20C,D, Figure 21C,D and Figure 22C,D). These windows were also helpful in the visualization of various air sacs, which were joined by connective tissue with adjacent organs or muscles (Figure 4C,D, Figure 5C,D, Figure 6C,D, Figure 7C,D, Figure 8C,D, Figure 9C,D, Figure 10C,D, Figure 11C,D, Figure 12C,D, Figure 13C,D, Figure 14C,D, Figure 15C,D, Figure 16C,D, Figure 17C,D, Figure 18C,D, Figure 19C,D and Figure 20C,D). In addition, this technology allowed to clarify structures from the genitourinary system. The three cranial divisions were clearly distinguished following a dorsal position in the coelomic cavity (Figure 11C,D, Figure 12C,D, Figure 13C,D, Figure 14C,D, Figure 15C,D, Figure 16C,D, Figure 17C,D, Figure 18C,D and Figure 19C,D). Lateroventrally to these divisions, the left ovary was appreciable (Figure 13C,D, Figure 14C,D, Figure 15C,D, Figure 16C,D, Figure 17C,D, Figure 18C,D and Figure 19C,D).

Finally, through tomographic studies carried out on the coelomic cavity of gulls, three-dimensional reconstruction models were generated using Maximum Intensity Projection (MIP) techniques. MIP reconstructions allowed clear visualization of the course of the trachea and the distribution of the gull air sacs (Figure 23B).

## 4. Discussion

Gulls, like other avian species, exhibit a highly specialized anatomical organization that differs markedly from that of mammals. In these birds, the absence of a distinct anatomical separation between the thoracic and abdominal compartments results in the formation of a single coelomic cavity containing the organs of the cardiovascular, respiratory, digestive, urinary, and reproductive systems [4,5,6,7,8,14,15,16,17]. The present study expands the currently limited anatomical information available for gulls by providing a detailed cross-sectional and computed tomography characterization of the coelomic cavity. To the authors’ knowledge, there are currently no published CT-based anatomical descriptions of the coelomic cavity in gull species. Nevertheless, similar imaging approaches have previously been applied to several avian species commonly encountered in zoological and clinical medicine, including the domestic pigeon, the toco toucan, and the grey parrot [5,18,19].

Within this anatomical context, these findings may also contribute to a broader understanding of the morphological organization of marine birds. Seabirds may show anatomical features related to flight, body mass distribution, and the organization of the respiratory system, many of which are reflected in the organization of the coelomic cavity and air sac system. Comparable anatomical patterns have been previously described in species such as the Atlantic puffin [3] and Cory’s shearwater [11], supporting the existence of several shared anatomical characteristics among marine avian taxa.

Conventionally, diagnostic evaluation in avian medicine has relied primarily on imaging modalities such as conventional radiography and ultrasonography [20]. Although radiography remains the most widely used first-line imaging technique because of its rapid acquisition, accessibility, and usefulness in the assessment of skeletal trauma, foreign bodies, and major coelomic alterations, the marked superimposition of anatomical structures and the limited serosal contrast characteristic of avian anatomy considerably restrict accurate evaluation of the coelomic cavity [5]. Ultrasonography may provide additional information regarding soft tissue structures, coelomic effusions, and image-guided procedures; however, its diagnostic applicability in birds is frequently limited by the extensive air sac system, restricted acoustic windows, and operator dependency [6,20]. In contrast, CT imaging allowed detailed visualization of the spatial organization and anatomical relationships of the coelomic organs in the present study, facilitating a more comprehensive evaluation of the avian coelomic cavity. Nonetheless, it also presents several important limitations, including the high cost of the equipment, the elevated procedural expenses, and the frequent requirement for sedation or physical restraint in avian patients. Consequently, the use of CT is generally reserved for selected clinical indications or for birds of particularly high individual value, such as breeding or falconry birds [4,5]. Owing to these practical constraints, conventional imaging techniques continue to represent the most employed diagnostic tools in routine avian practice because of their rapid execution, lower cost, and widespread accessibility.

Anatomical dissections and transverse sections were essential for the interpretation of the corresponding CT images. This combined approach improved the recognition of small or poorly contrasted structures and provided a more reliable topographic framework for assessing the anatomical relationships among the coelomic organs. The dissected specimens were particularly useful for identifying structures that may be difficult to distinguish on CT alone. As reported in other seabirds, including the puffin and the Cory’s Shearwater [3,8], the liver was well identified with the CT. However, anatomical dissection was particularly useful for confirming differences in hepatic lobe size. In contrast, we did not observe the division of the left hepatic lobe in caudodorsal and caudoventral subdivisions as presented in other birds [14]. In the yellow-legged gull, the spleen was observed in close association with the visceral surface of the liver and was positioned medial to the ventriculus. This topographic arrangement differs from that reported in some other avian species, in which the spleen shows different relationships with the proventriculus, ventriculus, or adjacent digestive structures [4,14]. In addition, the spleen displayed a predominantly fusiform morphology, rather than the flattened triangular shape previously described in anatomical studies of seabirds [14]. These differences may reflect species-specific anatomical adaptations or normal interspecific variation among seabirds, highlighting the importance of establishing dedicated anatomical references for each species. Moreover, these observations emphasize the importance of species-specific anatomical references when interpreting coelomic CT images in birds. The anatomical preparations also facilitated the identification of selected endocrine structures, including the thyroid and parathyroid glands. The thyroid glands were located bilaterally at the thoracic inlet, in close association with the common carotid arteries and adjacent major vessels. At their caudal aspect, small, spherical, consistently dark structures were identified as the parathyroid glands. These glands appeared less in number than those described in other avian species [14]. Whether this finding represents true species-specific variation or reflects the limited sample size requires confirmation in future studies. Recognition of these endocrine structures also helped identify related vascular structures, including the jugular and subclavian veins, as well as the cranial venae cavae. However, some small or thin-walled structures were not consistently visible in the transverse or dorsal CT reconstructions, probably because of their reduced size, limited intrinsic contrast, and the slice interval used in the anatomical sections. Thus, some relevant components of the digestive system including the caeca were only discernible in the dissected images, which as happens in pigeons, where short and rudimentary [14,15]. The rudimentary caeca observed in the yellow-legged gull may be related to the reduced role of cecal fermentation in species with predominantly carnivorous or omnivorous diets, although further comparative studies would be required to confirm this functional interpretation. Similar anatomical imaging studies in birds have shown that the correlation between gross anatomical preparations, sectional anatomy, and CT images is particularly valuable for interpreting complex body cavities and establishing species-specific anatomical references. A similar approach has been successfully applied in psittacines, toco toucans, parrots, Atlantic puffins, and Cory’s shearwaters [3,4,5,11,18,21,22]. In this context, the present study provides a species-specific anatomical reference that may support both comparative anatomical studies and the interpretation of diagnostic CT examinations in gulls.

In the present study, transverse and dorsal CT images of the coelomic cavity were evaluated using pulmonary, bone and soft tissue window settings. In addition to the anatomical correlation, the use of different CT window settings further improved image interpretation. The pulmonary window was particularly useful for differentiating the respiratory tract and for delineating air-containing structures and adjacent anatomical boundaries. It allowed clearer visualization of the lung parenchyma, primary bronchi, and air sacs, as well as improved delineation of aerated regions within the coelomic cavity. In contrast, the soft tissue window provided better contrast for the identification of parenchymal organs, including the liver, spleen, gastrointestinal tract, kidneys, and reproductive structures. The bone window was mainly valuable for assessing the osseous limits of the coelomic cavity, including the vertebrae, ribs, sternum, and pelvic girdle, and for clarifying the spatial relationship between skeletal landmarks and adjacent soft tissue structures. Overall, the combined use of different CT window settings enhanced anatomical interpretation by optimizing the visualization of tissues with different radiological densities. In addition, OsiriX MIP reconstruction of the gull’s coelomic cavity allowed adequate distinction of the different air sinuses, including the cervical, clavicular, interclavicular, cranial and caudal thoracic air sacs, as well as the abdominal air sacs. It is particularly relevant since they are a substantial for infection or compromise by husbandry-related processes [14]. This technique has already been used to evaluate the extension of paranasal sinuses in dogs and felids [23,24,25], improving the diagnosis of pathologies affecting these sinuses.

This investigation presents several limitations that should be considered when interpreting the findings. First, the number of specimens examined was relatively small, which may not fully represent the anatomical variability present within the species. Second, all examinations were performed on cadaveric specimens, preventing the assessment of physiological factors such as organ perfusion, respiratory dynamics, and normal variation associated with cardiac and respiratory cycles. In addition, six specimens were frozen and subsequently thawed before CT acquisition, which may have introduced minor alterations in tissue characteristics and attenuation values compared with those observed in live animals. Third, contrast-enhanced CT studies were not performed, limiting the visualization and characterization of vascular structures and reducing the distinction between some adjacent soft tissues. Potential anatomical differences related to sex, age, body condition, or reproductive status were also not specifically evaluated. Finally, the atlas was developed using clinically normal adult individuals and therefore does not address the appearance of pathological conditions that may modify the topographic relationships of coelomic organs. Despite these limitations, the combined use of gross anatomy, transverse sections, and CT imaging provides a comprehensive anatomical reference that may facilitate image interpretation and support future clinical and comparative studies in seabirds.

## 5. Conclusions

This study provides the first comprehensive cross-sectional and computed tomographic atlas of the coelomic cavity in the Yellow-legged Gull (*Larus michahellis*). Major respiratory, cardiovascular, digestive, urinary, and reproductive structures were identified and correlated with their CT appearance, allowing detailed characterization of their topographic relationships. The findings demonstrate the value of CT, particularly when evaluated using pulmonary, soft tissue, and bone windows, for the anatomical assessment of the avian coelomic cavity. The atlas established here provides a species-specific anatomical reference that may facilitate CT interpretation and improve the diagnosis of coelomic disorders in clinical, rehabilitation, and research settings. Nevertheless, further studies including a larger number of adult specimens of both sexes are warranted to investigate potential anatomical variability and to support the broader application of these imaging techniques.

## Figures and Tables

**Table 1:** Main biological characteristics of yellow-legged gulls.

Bird	Weight (g)	Body Length (cm)
1	957.4	54.5
2	663.3	46.5
3	776.9	40
4	785.1	41.3
5	631.9	40.2
6	673.5	43.5
7	807.2	44.5
8	665.4	39.5

## Data Availability

The information is available at https://accedacris.ulpgc.es (accessed on 29 July 2026).
